# DiffPaSS—high-performance differentiable pairing of protein sequences using soft scores

**DOI:** 10.1093/bioinformatics/btae738

**Published:** 2024-12-13

**Authors:** Umberto Lupo, Damiano Sgarbossa, Martina Milighetti, Anne-Florence Bitbol

**Affiliations:** Institute of Bioengineering, School of Life Sciences, École Polytechnique Fédérale de Lausanne (EPFL), Lausanne CH-1015, Switzerland; SIB Swiss Institute of Bioinformatics, Lausanne CH-1015, Switzerland; Institute of Bioengineering, School of Life Sciences, École Polytechnique Fédérale de Lausanne (EPFL), Lausanne CH-1015, Switzerland; SIB Swiss Institute of Bioinformatics, Lausanne CH-1015, Switzerland; Division of Infection and Immunity, University College London, London WC1E 6BT, United Kingdom; Cancer Institute, University College London, London WC1E 6DD, United Kingdom; Institute of Bioengineering, School of Life Sciences, École Polytechnique Fédérale de Lausanne (EPFL), Lausanne CH-1015, Switzerland; SIB Swiss Institute of Bioinformatics, Lausanne CH-1015, Switzerland

## Abstract

**Motivation:**

Identifying interacting partners from two sets of protein sequences has important applications in computational biology. Interacting partners share similarities across species due to their common evolutionary history, and feature correlations in amino acid usage due to the need to maintain complementary interaction interfaces. Thus, the problem of finding interacting pairs can be formulated as searching for a pairing of sequences that maximizes a sequence similarity or a coevolution score. Several methods have been developed to address this problem, applying different approximate optimization methods to different scores.

**Results:**

We introduce **Diff**erentiable **Pa**iring using **S**oft **S**cores (DiffPaSS), a differentiable framework for flexible, fast, and hyperparameter-free optimization for pairing interacting biological sequences, which can be applied to a wide variety of scores. We apply it to a benchmark prokaryotic dataset, using mutual information and neighbor graph alignment scores. DiffPaSS outperforms existing algorithms for optimizing the same scores. We demonstrate the usefulness of our paired alignments for the prediction of protein complex structure. DiffPaSS does not require sequences to be aligned, and we also apply it to nonaligned sequences from T-cell receptors.

**Availability and implementation:**

A PyTorch implementation and installable Python package are available at https://github.com/Bitbol-Lab/DiffPaSS.

## 1 Introduction

Identifying which proteins interact together, using their sequence data alone, is an important and combinatorially difficult task. Mapping the network of protein–protein interactions, and predicting the 3D structures of individual protein complexes, often requires determining which sequences are functional interaction partners among the paralogous proteins of two families. In the case of specific one-to-one interactions, this problem can be formulated as looking for a permutation of the sequences one family with respect to those of the other within each species. Sequence similarity-based scores (Goh and Cohen 2002, Gertz *et al.* 2003, Ramani and Marcotte 2003, Izarzugaza *et al.* 2006, Tillier *et al.* 2006, Izarzugaza *et al.* 2008, Tillier and Charlebois 2009, Bradde *et al.* 2010, Hajirasouliha *et al.* 2012, El-Kebir *et al.* 2013) and coevolution-based ones (Burger and van Nimwegen 2008, Bitbol *et al.* 2016, Gueudre *et al.* 2016, Bitbol 2018), as well as a method combining both ingredients (Gandarilla-Pérez *et al.* 2023) have been proposed to tackle this problem.

Sequence similarity is employed because when two proteins interact in one species, and possess close homologs in another species, then these homologs are likely to also interact. More generally, interacting protein families share a similar evolutionary history (Pazos and Valencia 2001, Ochoa and Pazos 2010, Ochoa *et al.* 2015), leading to sequence similarity-based pairing methods (Goh and Cohen 2002, Gertz *et al.* 2003, Ramani and Marcotte 2003, Izarzugaza *et al.* 2006, Tillier *et al.* 2006, Izarzugaza *et al.* 2008, Tillier and Charlebois 2009, Bradde *et al.* 2010, Hajirasouliha *et al.* 2012, El-Kebir *et al.* 2013). The use of neighbor graph alignment (Bradde *et al.* 2010) or of orthology determined by closest reciprocal hits for pairing interaction partners (Cong *et al.* 2019, Evans *et al.* 2021, Green *et al.* 2021, Humphreys *et al.* 2021) also relies on this idea.

The idea underlying coevolution-based methods for pairing interacting sequences is that amino acids that are in contact at the interface between two interaction partners need to maintain physico-chemical complementarity through evolution, which gives rise to correlations in amino-acid usage between interacting proteins (Burger and van Nimwegen 2008, Weigt *et al.* 2009, Bitbol *et al.* 2016, Gueudre *et al.* 2016, Bitbol 2018). Mutual information (Dunn *et al.* 2008) and pairwise maximum entropy models (Schug *et al.* 2009, Weigt *et al.* 2009, Marks *et al.* 2011, Morcos *et al.* 2011, Sułkowska *et al.* 2012, Mann *et al.* 2014) can reveal such coevolution both within a protein sequence and between the sequences of interacting partners. Additional correlations come from the shared evolutionary history of interacting partners (Marmier *et al.* 2019, Gerardos *et al.* 2022). Thus, permutations maximizing coevolution scores are expected to encode correct interactions (Bitbol *et al.* 2016, Bitbol 2018, Gandarilla-Pérez *et al.* 2023). Coevolution-based approaches require large and diverse multiple sequence alignments (MSAs) to perform well, which limits their applicability. More recently, scores coming from protein language models have also been proposed (Chen *et al.* 2023, Lupo *et al.* 2024). In particular, we proposed a method that outperforms traditional coevolution-based methods for shallow MSAs, comprising few sequences. However, this method is computationally intensive, and memory requirements limit its applicability to large MSAs.

We present **Diff**erentiable **Pa**iring using **S**oft **S**cores (DiffPaSS), a family of flexible, fast and hyperparameter-free algorithms for pairing interacting sequences among the paralogs of two protein families. DiffPaSS optimizes smooth extensions of coevolution or similarity scores to “soft” permutations of the input sequences, using gradient methods. It can be used to optimize any score, including coevolution scores and sequence similarity scores. Strong optima are reached thanks to a novel bootstrap technique, motivated by heuristic insights into this smooth optimization process. When using inter-chain mutual information (MI) between two MSAs as the score to be maximized, DiffPaSS outperforms existing coevolution- and sequence similarity-based pairing methods on difficult benchmarks composed of small MSAs from ubiquitous interacting prokaryotic systems. Compared to the protein language model based method DiffPALM (Lupo *et al.* 2024), DiffPaSS more rapidly produces paired alignments that can be used as input to AlphaFold-Multimer (Evans *et al.* 2021), in order to predict the 3D structure of protein complexes. We show promising results in this direction, for some eukaryotic complexes where the default AlphaFold-Multimer settings do not provide good performance. DiffPaSS is a general method that is not restricted to coevolution scores. In particular, it can be used to pair sequences by aligning their similarity graphs. We demonstrate that DiffPaSS outperforms a Monte Carlo simulated annealing method for graph alignment-based pairing of interacting partners on our benchmark prokaryotic data. Importantly, DiffPaSS graph alignment can be used even when reliable MSAs are not available. We show that it outperforms Monte Carlo graph alignment on the problem of pairing nonaligned sequences of T-cell receptor (TCR) CDR3*α* and CDR3*β* loops.

## 2 Materials and methods

### 2.1 Problem and general approach

#### 2.1.1 Pairing interacting protein sequences

Consider two protein families A and B that interact together, and the associated MSAs $M_{A}$ and $M_{B}$. Each of them is partitioned into *K* species, and we denote by *N_k_* the number of sequences in species *k*, with k=1,…,K. In practice, the number of members of family A and of family B in a species is often different. In this case, *N_k_* is the largest of these two numbers, and we add sequences made entirely of gap symbols to the MSA with fewer sequences in that species, as in Lupo *et al.* (2024).

Our goal is to pair the proteins of family A with their interacting partners from family B within each species. We assume for simplicity that interactions are one-to-one within each species. This is the first and main problem addressed by our method, and is commonly known as the paralog matching problem, as the within-species protein variants are often paralogs.

Our method also extends to related matching problems involving nonaligned sequences. This will be discussed below, with an application to variable regions of T-cell receptors, where alignment is challenging. Then, $M_{A}$ and $M_{B}$ are ordered collections of nonaligned amino-acid sequences instead of MSAs.

#### 2.1.2 Formalization

Let S be a score function of two MSAs (or ordered collections of nonaligned sequences) $M_{A}$ and $M_{B}$, which is sensitive to the relative ordering of the rows of $M_{A}$ with respect to those of $M_{B}$. Our matching problems can be formalized as searching for a permutation *π* of the rows of $M_{A}$ which maximizes $S(\pi (M_{A}),M_{B})$, denoted by S(π) for brevity. The permutation *π* should operate within each species and not across them, since interactions occur within a species. There are $\prod_{k=1}^{K}N_{k}!$ permutations satisfying this constraint, which usually renders unfeasible the brute-force approach of scoring all of them and picking the one with the largest score.

#### 2.1.3 General approach

Optimizing a score S across permutations is a discrete problem, but we propose an approximate differentiable formulation. Briefly, we first construct a differentiable extension $\hat{S}$ of S from permutation matrices to a larger space of matrices. This larger space comprises square matrices with nonnegative entries and whose all rows and columns (approximately) sum to 1. In what follows, we refer to such matrices as “soft permutations,” and to true permutation matrices as “hard permutations.” Using real square matrices, referred to as “parameterization matrices” X, a method (Mena *et al.* 2018) based on the Sinkhorn operator (Sinkhorn 1964) allows to smoothly navigate the space of soft permutations while keeping track of the “nearest” hard permutations (for more details, see the Supplementary Material). When this navigation is guided by gradient ascent applied to the extended score function $\hat{S}$, these hard permutations provide candidate solutions to the original problem.

In general, however, this differentiable optimization problem for $\hat{S}$ may have several local optima. Besides, depending on the precise way in which the discrete score S is extended to a differentiable score $\hat{S}$ for soft permutations, optimal soft permutations for $\hat{S}$ may be too distant from any hard permutation to approximate a well-scoring hard permutation for the original problem. Indeed, we found empirically, for a variety of scores, extensions, and random initializations, that naïvely applying the procedure above often yielded hard permutations with sub-optimal scores, even after several gradient steps. Nevertheless, we empirically found the following to be true for a wide class of scores and extensions thereof:

1. When gradient ascent is initialized so that the initial soft permutation for each species is “as soft as possible”—i.e. it is a square matrix with all entries equal to the same positive value, and normalized rows and columns—the first gradient step leads to a nearby *hard* permutation with significantly higher score than given by random expectation. This point is illustrated in Supplementary Fig. S1.
2. Pairing performance increases if some sequences are correctly paired and excluded from gradient optimization, while being included in the calculation of S and $\hat{S}$. As the size of this fixed context is increased, pairing performance for the remaining sequences increases. This point is consistent with previous approaches using other methods (Bitbol *et al.* 2016, Bitbol 2018, Gandarilla-Pérez *et al.* 2023, Lupo *et al.* 2024).

**Figure 1.**
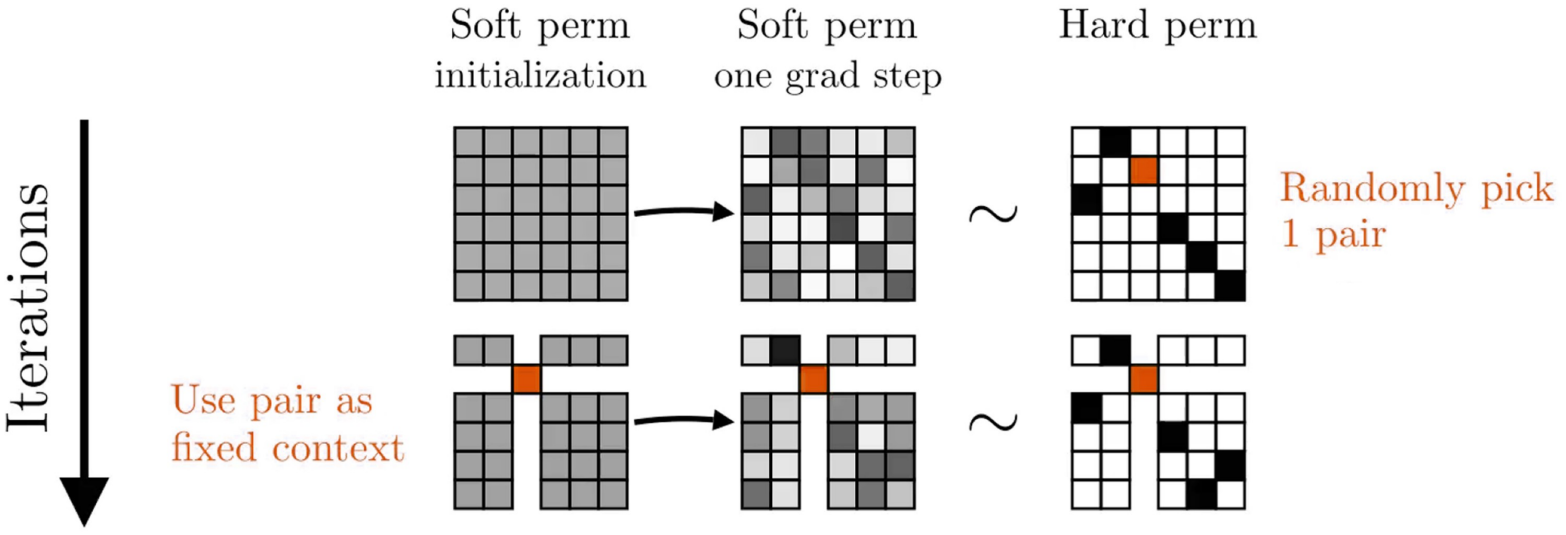
First two iterations of a DiffPaSS bootstrap. In this example, we pair sequences in a single species of size 6. Matrix entries are represented as shades of gray—ranging from white (when the entry is 0) to black (when the entry is 1). A specific pair, with entry highlighted in orange (dark gray in grayscale) in the hard permutation matrix, is randomly sampled at the end of an iteration, and then used as a fixed pair in the next iteration (its row and column are then shown as white without borders). A tilde (∼) between a soft and a hard permutation indicates that the soft permutation is “close” to the hard permutation, see Supplementary Equation (S2). An animation illustrating the full DiffPaSS bootstrap in this example is available at https://www.youtube.com/watch?v=G2rV4ldgTIY.

Together, these considerations motivated us to develop a bootstrapped approach to differentiable pairing, that we call DiffPaSS. Briefly, after a single gradient step using the procedure outlined above and the initialization described in point 1 above, a number *n* of pairings from the corresponding hard permutation is sampled uniformly at random, and used as fixed context in another run of gradient optimization using a single gradient step. The number *n* is gradually increased from 1 to the size of the collections of sequences to be paired, with a step size Δn, whose default value is one. Figure 1 illustrates the first two iterations of this method. See the Supplementary Material for more details. Note that in this bootstrap process, it is possible that some incorrect pairs are selected to be used as fixed context. This can also happen in existing methods such as IPA (Bitbol *et al.* 2016, Bitbol 2018), where it was found not to be too detrimental in practice, partly because coevolutionary signal from correct pairs adds constructively, while noise from incorrect pairs does not (Gandarilla-Pérez *et al.* 2020).

Using a single gradient step starting from our special initializations makes each step of the DiffPaSS bootstrap independent of gradient optimization hyperparameters such as learning rate and regularization strength. It also makes it independent of the two hyperparameters needed to define the (truncated) Sinkhorn operator, see Supplementary Material. Thus, in the DiffPaSS bootstrapped optimization process, the only parameter that can be tuned is the step size Δn.

### 2.2 Mutual-information-based scores for MSAs

When $M_{A}$ and $M_{B}$ are MSAs, denoting by $M_{A,i}$ the *i*-th column of $M_{A}$ (and analogously for $M_{B}$), we define the *inter-chain mutual information* score $S_{\text{MI}}(M_{A},M_{B})$ by summing the mutual information estimates of all pairs of columns composed of one column in $M_{A}$ and one in $M_{B}$ (Bitbol 2018):

(1) $$\begin{array}{cc} & S_{\text{MI}}(M_{A},M_{B})=\sum_{i,j}I(M_{A,i};M_{B,j})\\ & =\sum_{i,j}[H(M_{A,i})+H(M_{B,j})-H(M_{A,i},M_{B,j})], \end{array}$$

where I(·;·) denotes the mutual information between two random variables, and H denotes the Shannon entropy. In practice, we replace these information quantities by their plug-in estimates, i.e. we use observed frequencies instead of probabilities (see Bitbol 2018). Since, for any *i* and permutation *π*, $H(M_{A,i})=H(\pi {\left(M_{A}\right)}_{i})$, maximizing $S_{\text{MI}}(\pi )$ over permutations *π* is equivalent to minimizing the *inter-chain two-body entropy loss* $L_{2\text{BE}}(\pi )$ over permutations, with $L_{2\text{BE}}(\pi )$ defined as

(2) $$L_{2\text{BE}}(\pi )=\sum_{i,j}H(\pi {\left(M_{A}\right)}_{i},M_{B,j}).$$

We define a smooth extension ${\hat{L}}_{2\text{BE}}$ of $L_{2\text{BE}}$ to soft permutations as follows. First, we represent the MSAs using one-hot encoding. Namely, let $m_{A,i,n}$ denote the one-hot vector corresponding to row (i.e. sequence) *n* and column (i.e. site) *i* in $M_{A}$—and similarly for $M_{B}$. The matrix of observed counts of all joint amino-acid states at the column pair (*i*, *j*) can then be computed as $\sum_{n}m_{A,i,n}⊗m_{B,j,n}$, where ⊗ denotes vector outer product. As this expression is well-defined and smooth for pairs of arbitrary vectors, it yields a smooth extension of counts and frequencies provided that all vector entries are nonnegative. This leads to a smooth extension $\hat{H}(\cdot ,\cdot )$ of the two-body entropy H(·,·). In our case, for a soft permutation $\hat{\pi }$ represented as a matrix $\hat{P}$, we introduce a *soft MSA* as $\hat{\pi }(M_{A})=\hat{P}M_{A}$, where $M_{A}$ is the representation of $M_{A}$ as a tensor with an additional one-hot dimension. We thus define the following smooth extension ${\hat{L}}_{2\text{BE}}$ of the inter-chain two-body entropy loss $L_{2\text{BE}}$:

(3) $${\hat{L}}_{2\text{BE}}(\hat{\pi })=\sum_{i,j}\hat{H}({\left(\hat{P}M_{A}\right)}_{i},M_{B,j}).$$

### 2.3 Other scores

So far, we motivated the DiffPaSS bootstrap framework using the data structure of MSAs and the MI between MSA columns. This was based on the observation (Bitbol 2018) that MI contains useful signal for matching paralogs between interacting protein families. However, a variety of other scores can also be used for paralog matching and for more general pairing problems, including scores based on sequence similarities, orthology, and phylogeny (Goh and Cohen 2002, Gertz *et al.* 2003, Ramani and Marcotte 2003, Izarzugaza *et al.* 2006, Tillier *et al.* 2006, Izarzugaza *et al.* 2008, Tillier and Charlebois 2009, Bradde *et al.* 2010, Hajirasouliha *et al.* 2012, El-Kebir *et al.* 2013). In some cases, these alternative scores are available even when alignments are not easy to construct or even meaningful. Importantly, the DiffPaSS framework is a general approach that can be applied to different scores. As an example, we extend the DiffPaSS framework to optimize graph alignment scores, and we consider applications to both aligned and nonaligned sequences.

### 2.3.1 Graph alignment scores

Let us consider two ordered collections of sequences $M_{A}$ and $M_{B}$. Let us define a weighted graph $G_{A}$ (resp. $G_{B}$), whose nodes represent the sequences in $M_{A}$ (resp. $M_{B}$), and whose pairwise weights are stored in a matrix $W_{A}$ (resp. $W_{B}$). Graph alignment (GA) can be performed between $G_{A}$ and $G_{B}$ using a variety of loss functions (Petric Maretic *et al.* 2019, Maretic *et al.* 2022a,b; Gandarilla-Pérez *et al.* 2023). When the GA loss function $L_{\text{GA}}(W_{A},W_{B})$ is differentiable, we propose the following variant of DiffPaSS: define ${\hat{L}}_{\text{GA}}(\hat{\pi })=L_{\text{GA}}(\hat{P}W_{A}{\hat{P}}^{T},W_{B})$, where $\hat{\pi }$ is a soft permutation encoded by the square matrix $\hat{P}$. This definition is natural in the special case where $\hat{\pi }$ is a hard permutation, as $\hat{P}W_{A}{\hat{P}}^{T}$ is then the matrix obtained from $W_{A}$ by permuting its rows and columns by $\hat{\pi }$. Then, we perform a DiffPaSS bootstrap procedure (see above), using $\hat{L}$ as the differentiable loss.

Here, as in references (Bradde *et al.* 2010, Gandarilla-Pérez *et al.* 2023), we align *k*-nearest neighbor graphs. Specifically, we use pairwise weight matrices W constructed from ordered collections $M={\left(s_{i}\right)}_{i}$ of sequences as follows. Considering a symmetric distance (or dissimilarity) metric *d* between sequences and an integer *k *>* *1, we define the (*i*, *j*)-th entry of W as

(4) Wij={e−d(si,sj)/D2if si is among the k nearest neighbors of sj, or vice–versa,0otherwise.

Here, *D* is the average distance (over all sequences in M) of the *k*-th nearest neighbor sequence. As distance metric, we use Hamming distances if the sequences are aligned, and edit distances otherwise. We set *k *=* *20 or *k *=* *30. Finally, as in references (Bradde *et al.* 2010, Gandarilla-Pérez *et al.* 2023), we use

(5) $$L_{\text{GA}}(W_{A},W_{B})=-\sum_{i}\sum_{j>i}{\left(W_{A}\right)}_{ij}{\left(W_{B}\right)}_{ij}$$

as a GA loss function.

### 2.4 Robust pairs and iterative variants of DiffPaSS

We empirically found that, when running a DiffPaSS bootstrap, some sequence pairs are found by all the hard permutations explored. We call these *robust pairs*, and notice that they tend to have high precision. This is illustrated in Supplementary Fig. S2 for a benchmark prokaryotic dataset described in Supplementary Section S2. This suggests that they can be used as a starting set $F_{\text{AB}}$ of fixed pairs in another run of DiffPaSS, where further fixed pairs are (randomly) chosen from the remaining sequences, see above. This process can be repeated several times by adding new robust pairs, found among the remaining sequences, to the set $F_{\text{AB}}$. We call this iterative procedure DiffPaSS-IPA [“Iterative Pairing Algorithm”, following (Bitbol *et al.* 2016, Bitbol 2018)]. The final output of DiffPaSS-IPA is the hard permutation with lowest observed loss across all IPA runs. In practice, for all IPA variants of DiffPaSS, we use $N_{\text{IPA}}=3$ iterations here.

## 3 Results

### 3.1 DiffPaSS accurately and efficiently pairs paralogs using MI

#### 3.1.1 DiffPaSS-MI pairs interacting sequences more accurately than other MI-based methods

**Figure 2.**
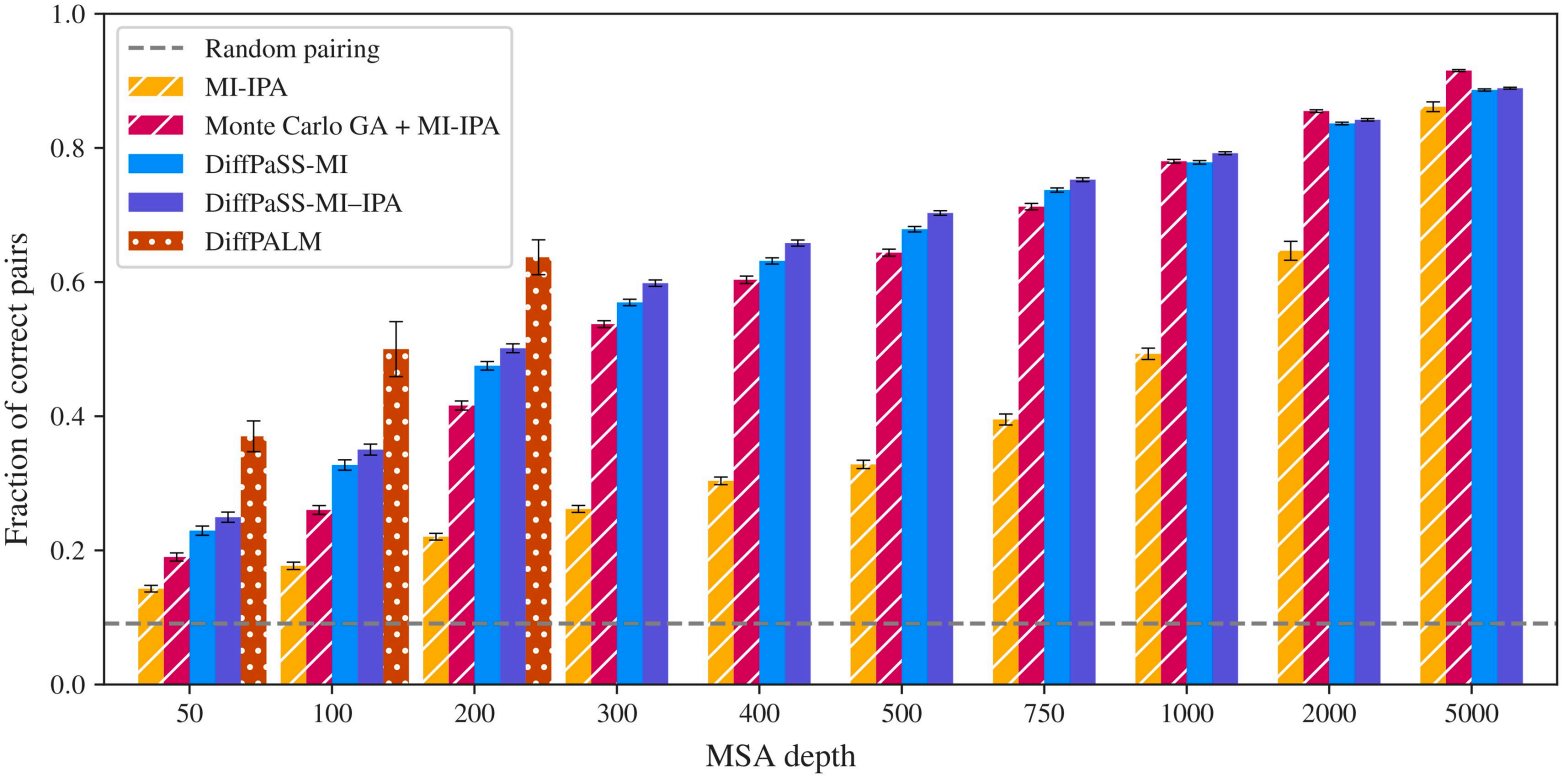
Performance of coevolution-based pairing methods on HK-RR MSAs. The performance of DiffPaSS-MI and DiffPaSS-MI-IPA is compared to that of MI-IPA (Bitbol 2018), alone or combined with Monte Carlo GA (Gandarilla-Pérez *et al.* 2023), and to that of DiffPALM (Lupo *et al.* 2024), for HK-RR MSAs with different depths. The HK-RR dataset is described in Supplementary Section S2. With all methods, a full one-to-one within-species pairing is produced, and performance is measured as the fraction of correct pairs among all predicted pairs. For the combined Monte Carlo GA and MI-IPA method, we used the same settings as Gandarilla-Pérez *et al.* (2023). Results for DiffPALM are taken from Lupo *et al.* (2024).

Let us first consider coevolution-based pairing of partners from the MSAs of two interacting protein families. How does our differentiable pairing method compare to existing discrete pairing methods? To address this question, we test DiffPaSS and DiffPaSS-IPA, using the total inter-chain MI as a score (see Section 2), on a benchmark dataset composed of ubiquitous prokaryotic proteins from two-component signaling systems (Barakat *et al.* 2009, 2011), which enable bacteria to sense and respond to environment signals. This dataset comprises cognate pairs of histidine kinases (HKs) and response regulators (RRs) determined using genome proximity, see Supplementary Section S2. These proteins feature high specificity and generally interact one-to-one (Laub and Goulian 2007, Cheng *et al.* 2014). We blind all partnerships and we compare different coevolution-based methods on the task of pairing these interacting sequences within each species, without giving any known paired sequences as input. Figure 2 shows that both DiffPaSS and DiffPaSS-IPA significantly outperform the MI-IPA algorithm (Bitbol 2018), which performs a discrete approximate maximization of the same MI score. The gain of performance obtained by using DiffPaSS instead of MI-IPA is particularly remarkable for relatively shallow MSAs, up to ∼2000 sequences deep. Recall that MI-IPA is quite data-thirsty (Bitbol 2018). Furthermore, Fig. 2 shows that, for MSAs up to depth 750 (resp. 1000), DiffPaSS (resp. DiffPaSS-IPA) outperforms a recent method which combines Monte-Carlo GA and MI-IPA (Gandarilla-Pérez *et al.* 2023). This is significant because this combined GA and MI-IPA method exploits both phylogenetic sequence similarity via GA and coevolution via MI, while here we only used MI in DiffPaSS. However, the combination of GA and MI-IPA slightly outperforms MI maximization (with DiffPaSS) for deeper MSAs (depths 2000 and 5000)—see Section 3.1.3 for additional information. Supplementary Figure S3 shows that, for deep alignments, the performance of DiffPaSS-MI is improved by combining it with GA, and becomes comparable with that of Monte Carlo GA combined with MI-IPA. Note that for shallow alignments, combining with GA improves the performance of MI-IPA, but not that of DiffPaSS-MI.

An important parameter of GA is the number *k* of nearest neighbors considered in the *k* nearest-neighbor (*k*NN) graphs that are aligned, see Equation (4). While in (Gandarilla-Pérez *et al.* 2023) it was optimized on the same HK-RR dataset as the one we use here, this was for MSAs of depth 5000. In Supplementary Fig. S4, we investigate the impact of *k* on the performance of MI-IPA combined with Monte Carlo GA for HK-RR MSAs of depth 100. We observe that smaller values of *k* lead to slightly better performances for these shallower MSAs, but that the performance of DiffPaSS-MI-IPA remains higher than that of MI-IPA combined with Monte Carlo GA for all values of *k* considered. Note that for deeper MSAs (depth 5000), Fig. 2 shows that all methods, including MI-IPA, perform well and obtain correct results for >80% of the pairs (Bitbol 2018, Gandarilla-Pérez *et al.* 2023).

While DiffPaSS-MI and MI-IPA are both based on MI, we recently introduced DiffPALM (Lupo *et al.* 2024), an approach that leverages the MSA-based protein language model MSA Transformer (Rao *et al.* 2021). Figure 2 shows that DiffPaSS does not reach the performance achieved by DiffPALM. However, DiffPaSS is several orders of magnitude faster (see below), and easily scales to much deeper MSA depths for which DiffPALM cannot be run due to memory limitations.

An attractive feature of the DiffPaSS bootstrap process is that the only hyperparameter that can be varied is the integer step size Δn controlling the number of fixed pairs added at each bootstrap step (see Section 2). By default, we choose a step size Δn=1. We found that it led to better performance of DiffPaSS-MI-IPA than using a step size Δn=2, see Supplementary Fig. S5. Note however that increasing Δn can lead to significant speed-ups.

#### 3.1.2 Extension to another benchmark prokaryotic dataset

So far, we tested DiffPaSS on the HK-RR benchmark. In Supplementary Fig. S6, we show the performance of DiffPaSS-MI-IPA on a dataset of ABC transporter proteins homologous to the *Escherichia coli* MALG-MALK pair of interacting proteins, see references (Bitbol *et al.* 2016, Bitbol 2018, Gandarilla-Pérez *et al.* 2023, Lupo *et al.* 2024), for MSAs of depth 100. We find that in this case, the performance of DiffPaSS-MI-IPA is comparable to that of MI-IPA combined with Monte Carlo GA for various values of *k*, and to that of DiffPALM. This confirms that the ability of DiffPaSS at predicting interacting pairs generalizes beyond the HK-RR case.

#### 3.1.3 DiffPaSS-MI is highly effective at extracting MI signal

**Figure 3.**
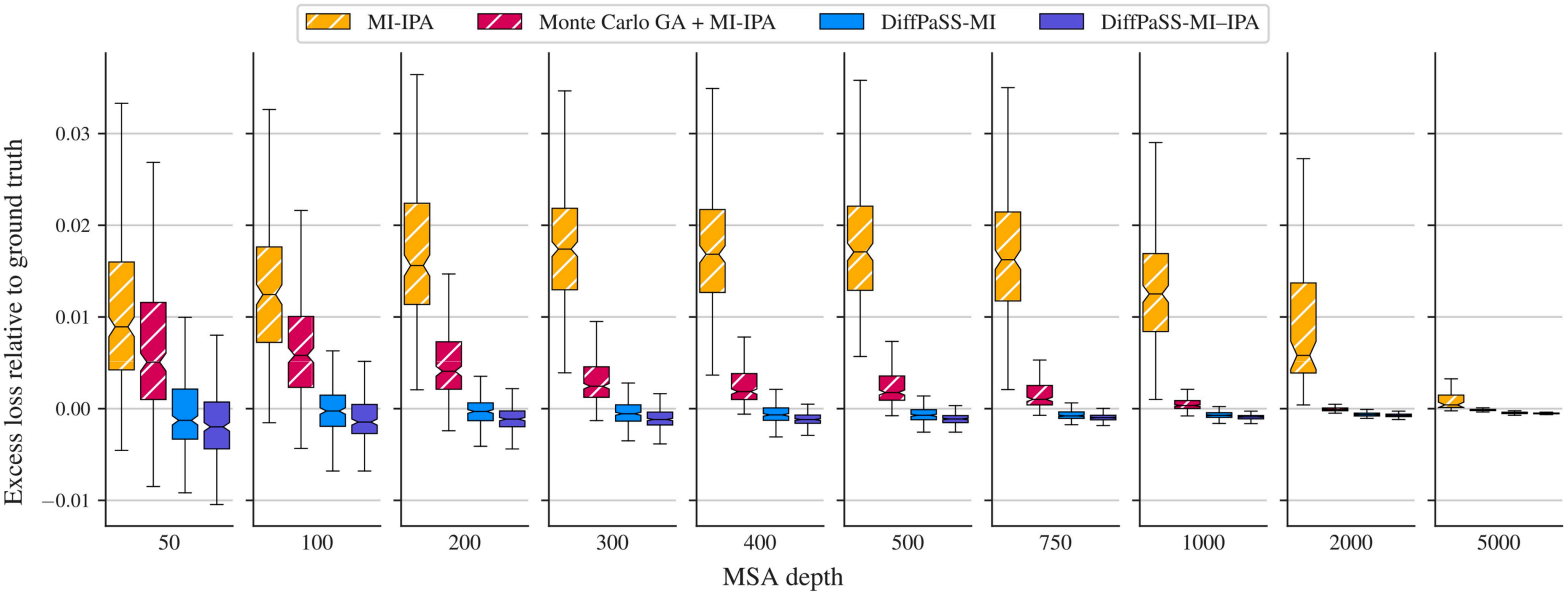
Inter-chain two-body entropy losses for predicted pairings. The distributions of the excess inter-chain two-body entropy losses of predicted pairings relative to ground-truth pairings are shown as box plots for several methods and several MSA depths, on the HK-RR dataset (same MSAs as in Fig. 2).

How does DiffPaSS achieve better performance than MI-IPA? It was shown in (Bitbol 2018) that, for relatively shallow MSAs, the approximate discrete optimization algorithm used to maximize inter-chain MI [Equation (1)] in the MI-IPA approach does not provide pairings that reach MI values as high as the ground-truth pairings. Thus motivated, we compare the inter-chain MI of the paired MSAs produced by DiffPaSS, and by other methods, to the MI of the ground-truth pairings. We use the difference between the inter-chain two-body entropy loss [Equation (2)] of a paired MSA and that of the ground-truth paired MSA. Indeed, this “excess loss” is equal to minus the difference between the corresponding inter-chain MIs, see Equations (1) and (2). Figure 3 shows the distribution of excess inter-chain two-body entropy losses for each method. We observe that the median MI of DiffPaSS(-IPA) final pairings is indistinguishable or higher than that of the ground-truth pairings for all MSA depths considered (as excess losses are positive). Furthermore, the distribution of excess losses across MSAs becomes very narrow for deeper MSAs, showing that a score very close to the ground-truth MI is then systematically obtained. Meanwhile, for shallow MSAs, MI-IPA and the combined Monte Carlo GA + MI-IPA methods yield paired MSAs that have a substantially lower MI than the ground-truth pairings. This improves for deeper MSAs, faster for the combined method than for MI-IPA [see also Bitbol (2018) for MI-IPA]. Note however that, for all MSA depths, the combined method yields paired MSAs with higher excess losses than DiffPaSS-MI(-IPA), even though it produces a higher fraction of correct pairs, on average, for the deepest MSAs (depths 2000 and 5000, see Fig. 2). This shows that, at least on this dataset, the inter-chain two-body entropy loss has some limitations for the pairing of interaction partners, in the sense that the correctly paired MSA has a somewhat higher loss (i.e. lower MI) than some slightly different ones. This might arise due to phylogenetic relationships between sequences. Indeed, exploiting sequence similarity and phylogeny via GA (Gandarilla-Pérez *et al.* 2023) provides additional information. Recall that Supplementary Fig. S3 shows that this information can be exploited when using DiffPaSS-MI as well as when using MI-IPA.

While our focus is here on comparing to the ground-truth pairings, the distributions of inter-chain two-body losses are shown in Supplementary Fig. S7, for all methods as well as for random pairings. This figure shows the amount of available MI signal for pairing in these datasets. Overall, these results demonstrate that DiffPaSS is extremely effective at extracting the available MI signal.

#### 3.1.4 DiffPaSS-MI identifies robust pairs

The combined method Monte Carlo GA + MI-IPA relies on identifying robust pairs which are predicted by multiple independent runs of GA and then using them as a training set of known pairs for MI-IPA. Supplementary Figure S2 compares these robust pairs with those that are identified by DiffPaSS-MI(-IPA), specifically the set $F_{\text{AB}}$ after 3 IPA iterations, see Section 2. It shows that, for relatively shallow MSAs, DiffPaSS-IPA typically identifies fewer correct pairs as robust than Monte Carlo GA, but its robust pairs are correct more often. For deeper MSAs, the number of correct robust pairs identified by DiffPaSS-MI-IPA sharply increases, while it plateaus for Monte Carlo GA.

#### 3.1.5 DiffPaSS-MI is substantially faster than other approaches

How does DiffPaSS compare to existing algorithms in terms of computational runtime? DiffPaSS can easily be run on a modern GPU, while MI-IPA and Monte Carlo GA + MI-IPA are CPU-only algorithms. Supplementary Figure S8 demonstrates that DiffPaSS-MI is considerably faster than Monte Carlo GA + MI-IPA across all the MSA depths we analyzed. DiffPALM (Lupo *et al.* 2024) (not shown in Supplementary Fig. S8) has much longer runtimes than both methods, taking e.g. over three orders of magnitudes longer than DiffPaSS to pair HK-RR MSAs of depth ∼50. Thus, a considerable asset of DiffPaSS is its rapidity, which makes it scalable to large datasets comprising many pairs of protein families.

### 3.2 DiffPaSS improves the structure prediction by AlphaFold-Multimer of some eukaryotic complexes

**Figure 4.**
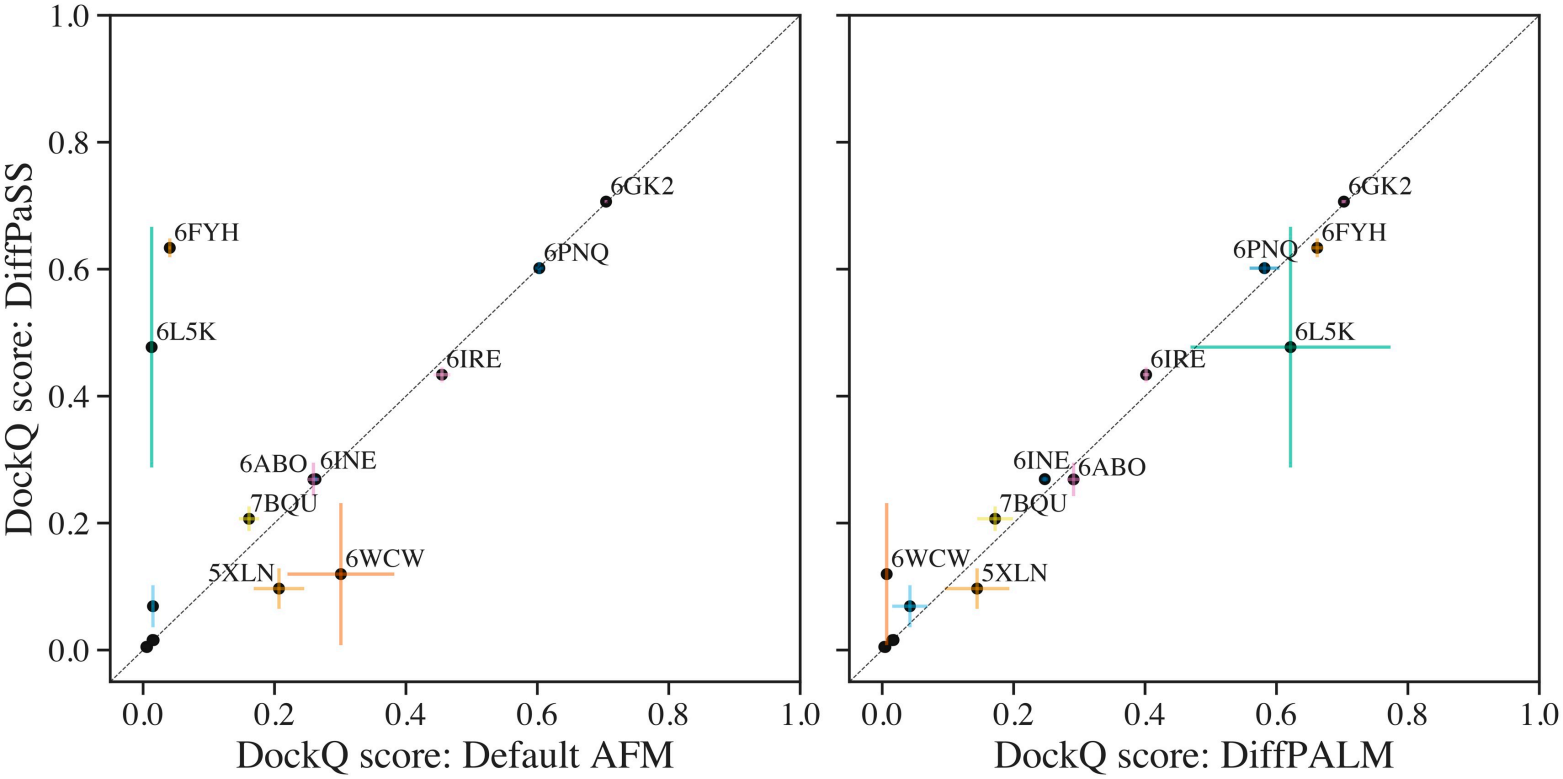
Performance of structure prediction by AFM using different MSA pairing methods. We report the performance of AFM, in terms of DockQ scores, for the 15 complexes considered in Lupo *et al.* (2024), using different pairing methods on the same MSAs. Left panel: DiffPaSS versus default AFM pairing. Right panel: DiffPaSS versus DiffPALM. For each complex, AFM is run five times, and the top predicted structure by AFM confidence is considered each time, yielding five predicted structures in total. DockQ scores are averaged over the five predictions and standard errors are shown as error bars. Points with DockQ below 0.1 are not labelled with their PDB ID for graphical reasons.

While genome proximity can often be used to pair interaction partners in prokaryotes, it is not the case in eukaryotes. Pairing correct interaction partners is thus a challenging problem in eukaryotes, which also often have many paralogs per species (Makarova *et al.* 2005) while eukaryotic-specific protein families generally have fewer total homologs and smaller diversity than in prokaryotes. Solving this problem has an important application to the prediction of protein complex structure. Indeed, AlphaFold-Multimer (AFM) (Evans *et al.* 2021) relies on paired MSAs (Evans *et al.* 2021, Bryant *et al.* 2022). Can DiffPaSS improve complex structure prediction by AFM (Evans *et al.* 2021) in eukaryotic complexes? As a first exploration of this question, we consider the same 15 eukaryotic complexes as in (Lupo *et al.* 2024), where improvements over the default AFM pairing methods were reported by pairing using the MSA-Transformer-based method DiffPALM (Rao *et al.* 2021). More information on these structures and on the AFM setup can be found in Supplementary Sections S2 and S3. Figure 4 compares the performance of AFM on these complexes, using three different pairing methods (default AFM, DiffPALM, and DiffPaSS-MI) on the same initial unpaired MSAs. We use the DockQ score, a widely used measure of quality for protein–protein docking (Basu and Wallner 2016), as a performance metric for complex structure prediction. More details are given in Supplementary Fig. S9, where we include the top 5 predicted structures of each run of AFM. AFM confidence scores for these predictions are also shown in Supplementary Fig. S10. These results show that DiffPaSS can improve complex structure prediction in some cases. Furthermore, our results are consistent with those we previously obtained with DiffPALM (Lupo *et al.* 2024): the two structures that are substantially improved are 6FYH and 6L5K.

### 3.3 DiffPaSS allows accurate graph alignment

**Figure 5.**
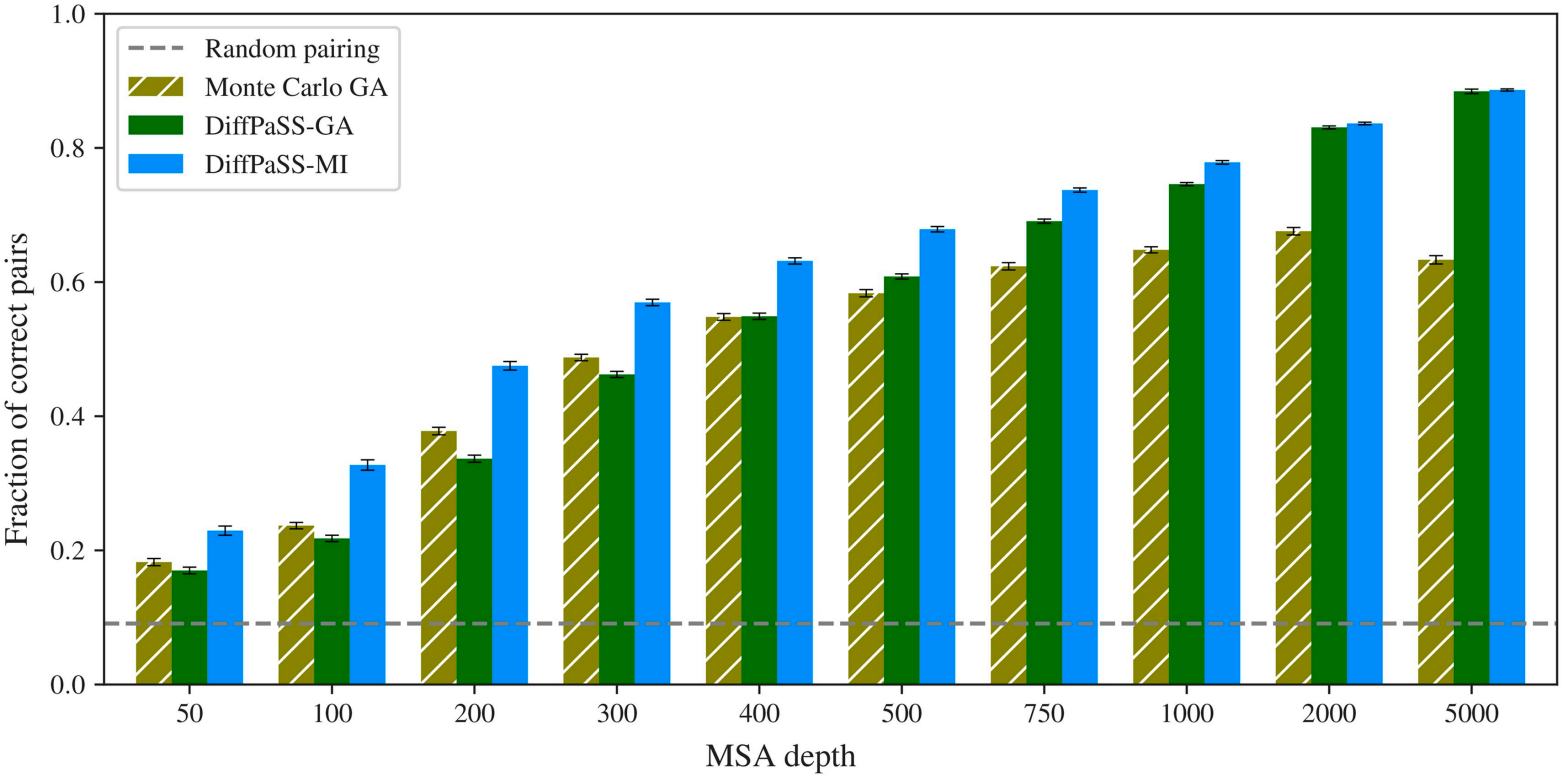
Performance of GA- or MI-based pairing methods on HK-RR MSAs. The fraction of correct pairs obtained is shown for DiffPaSS-GA and for the Monte Carlo-based GA implementation from Gandarilla-Pérez *et al.* (2023), on the same HK-RR MSAs as in Fig. 2. The results for DiffPaSS-MI from Fig. 2 are shown for comparison.

So far, we used DiffPaSS for the specific problem of paralog matching from the MSAs of two interacting protein families, using coevolution measured via MI. However, the optimization approach of DiffPaSS is general and can be applied to other scores, and to nonaligned sequences. In particular, it can be used for the problem of graph alignment, see Section 2. To assess how well DiffPaSS performs at this problem, we first return to our benchmark HK-RR dataset. Instead of the inter-chain MI, to predict pairs we now use the GA loss (Bradde *et al.* 2010, Gandarilla-Pérez *et al.* 2023), defined in Equation (5). Note that we use Hamming distances to define the weight matrices $W_{A}$ and $W_{B}$. Figure 5 shows that DiffPaSS-GA is competitive with the Monte Carlo simulated annealing algorithm in (Gandarilla-Pérez *et al.* 2023), which optimizes the same GA loss. More precisely, DiffPaSS-GA performs slightly less well than Monte Carlo GA for shallow MSAs, but better than it for deeper ones, where the GA problem becomes trickier as the neighbor graph contains more and more inter-species edges. We also compare DiffPaSS-GA with DiffPaSS-MI, and find that DiffPaSS-MI outperforms DiffPaSS-GA for shallow MSAs, but performance becomes similar for deeper ones.

Contrary to MI, GA does not require sequences to be aligned. Indeed, one can use a distance measure between sequences which does not involve aligning them. This opens the way to broader applications. An interesting one, which is close to the paralog matching problem studied here, regards pairing T cell receptor chains. Specifically, we consider collections of sequences from hypervariable CDR3*α* and CDR3*β* loops in TCRs binding to a fixed epitope, as in Milighetti *et al.* (2024). The task is to pair each *α* chain to its *β* chain within each human patient. Thus, here, patients play the part of species in our paralog matching problem. Our dataset of paired CDR3*α*-CDR3*β* loop sequences from TCRs is described in Supplementary Section S2. These collections of CDR3*α* and CDR3*β* sequences are difficult to align due to their hypervariability, motivating the choice of GA for this problem, using the GA loss in Equation (5) with weight matrices $W_{A}$ and $W_{B}$ defined using edit distances instead of Hamming distances [see Equation (4)]. Note also that here we use *k *=* *20 nearest neighbors, in order to compare our results with those from Milighetti *et al.* 2024, obtained using the same value of *k*. The differences between the losses obtained by DiffPaSS and those obtained by the Monte Carlo GA algorithm are shown in Supplementary Fig. S11. DiffPaSS achieves substantially lower losses than the Monte Carlo GA algorithm, on average, in the four datasets containing the largest numbers of sequences to pair (from ∼700 to ∼2000). On the other hand, it yields slightly higher losses than Monte Carlo GA, on average, for datasets with 500 pairs or fewer. The good performance of DiffPaSS-GA on larger datasets is consistent with our HK-RR results (Fig. 5).

## 4 Discussion

We introduced DiffPaSS, a framework to pair interacting partners among two collections of biological sequences. DiffPaSS uses a versatile and hyperparameter-free differentiable optimization method that can be applied to various scores. It outperforms existing discrete optimization methods for pairing paralogs using MI, most spectacularly in the regime of shallow alignments. Strikingly, on our benchmark datasets, DiffPaSS is able to extract all the MI signal available for pairing. DiffPaSS is computationally highly efficient, compared to existing discrete optimization methods and, by far, to DiffPALM (Lupo *et al.* 2024), our pairing method based on MSA Transformer. Thus, it has the potential be applied to large datasets. Our first explorations on a small set of eukaryotic protein complexes show that paired alignments produced by DiffPaSS may lead to improvements of complex structure prediction by AlphaFold-Multimer (Evans *et al.* 2021). DiffPaSS does not need to start from aligned sequences, thus opening the way to broader applications. Our results on pairing TCR chains show promise for the use of DiffPaSS to optimize scores that cannot be constructed from MSAs.

Since DiffPaSS shows promise for structural biology, it would be very interesting to apply it more broadly to protein complex structure prediction problems. Besides, applications to TCRs are also promising. We expect CDR3*α*-CDR3*β* paired sequences to show lower co-evolution signal than other interacting protein families. Indeed, they are generated at random and selected for their joint ability to bind a given antigen, rather than directly co-evolving. Development of similarity measures that can cluster TCRs with the same specificity in sequence space is ongoing (Thomas *et al.* 2014, Dash *et al.* 2017, Thakkar and Bailey-Kellogg 2019, Vujovic *et al.* 2020, Wu *et al.* 2024, Meysman *et al.* 2023, Nagano *et al.* 2024). Using DiffPaSS-GA with these similarity measures could allow testing of metrics specifically optimized to capture TCR co-specificity.

One limitation of DiffPaSS in its current formulation is that it assumes one-to-one pairings. It would be very interesting to extend it to pairing problems between collections of different sizes. To achieve this, one could e.g. replace the Sinkhorn operator with Dykstra’s operator, see (Maretic *et al.* 2022b).

Finally, despite DiffPaSS’s ability to extract all available MI signal from our benchmark dataset, we found that our MSA-Transformer–based pairing method DiffPALM outperforms DiffPaSS. This shows that MSA-based protein language models such as MSA Transformer truly capture more signal usable for pairing than MI.

## Supplementary Material

btae738_Supplementary_Data
