# Supplementary material for: DiffPaSS—high-performance differentiable pairing of protein sequences using soft scores
Source: Bioinformatics. 2024 Dec 13;41(1):btae738. doi: 10.1093/bioinformatics/btae738 (PMC11676329; doi:10.1093/bioinformatics/btae738)
Supplement: btae738_Supplementary_Data [file btae738_supplementary_data.pdf]

# Supplementary material for “DiffPaSS – High-performance differentiable pairing of protein sequences using soft scores”

Umberto Lupo[1,2,†], Damiano Sgarbossa[1,2], Martina Milighetti[3,4], Anne-Florence Bitbol[1,2,†]

**1** Institute of Bioengineering, School of Life Sciences, École Polytechnique Fédérale de Lausanne (EPFL), CH-1015 Lausanne, Switzerland
**2** SIB Swiss Institute of Bioinformatics, CH-1015 Lausanne, Switzerland
**3** Division of Infection and Immunity, University College London, London, United Kingdom
**4** Cancer Institute, University College London, London, United Kingdom
† Emails: umberto.lupo@epfl.ch, anne-florence.bitbol@epfl.ch

## Contents

**1 Detailed methods** **1**
1.1 Preliminaries . . . . . 1
1.2 Initialization and bootstrapped optimization . . . . . 2
1.3 Independence of number of Sinkhorn normalizations . . . . . 3

**2 Datasets** **4**

**3 General points on AlphaFold-Multimer (AFM)** **5**

**4 Supplementary figures** **5**

# 1 Detailed methods

In this section, we present a more detailed account of the methods developed in this work. We acknowledge that there are some redundancies with the main text methods. We retained them for the sake of completeness and readability of this Supplementary material.

## 1.1 Preliminaries

Let $\mathcal{M}_{\mathrm{A}}$ and $\mathcal{M}_{\mathrm{B}}$ be ordered collections of amino-acid sequences that are partitioned into $K$ groups, each of size $N_k$ where $k = 1, \ldots, K$. In the important special case where $\mathcal{M}_{\mathrm{A}}$ and $\mathcal{M}_{\mathrm{B}}$ are collections of proteins from two interacting protein families, the “groups” will be species, with species $k$ assumed to contain $N_k$ paralogous proteins in both families.

Let $\mathscr{S}$ be a score function of the two ordered collections. We would like to find a permutation $\pi$ of the entries in $\mathcal{M}_{\mathrm{A}}$ which maximises $\mathscr{S}(\pi(\mathcal{M}_{\mathrm{A}}), \mathcal{M}_{\mathrm{B}})$, under the constraint that $\pi$ does not send sequences from any one group into a different group. A priori, there are $\prod_{k=1}^{K} N_k!$ permutations satisfying this constraint. Note that we can equivalently describe this as the problem of finding $\mathscr{S}$-maximising one-to-one matchings between the sequences in $\mathcal{M}_{\mathrm{A}}$ and those in $\mathcal{M}_{\mathrm{B}}$. Since $\mathcal{M}_{\mathrm{A}}$ and $\mathcal{M}_{\mathrm{B}}$ will remain fixed, by abusing notation we denote $\mathscr{S}(\pi(\mathcal{M}_{\mathrm{A}}), \mathcal{M}_{\mathrm{B}})$ simply by $\mathscr{S}(\pi)$.

Let $N > 0$, and denote the set of permutation matrices of $N$ elements by $\mathcal{P}_N$. For any integer $\ell > 0$ and arbitrary $N \times N$ square matrix $X$, define the *$\ell$-truncated Sinkhorn operator*

$$S^{\ell}(X) = (\mathcal{C} \circ \mathcal{R})^{\ell}(\exp(X)) \tag{S1}$$

consisting of first applying the componentwise exponential function exp to $X$, and then iteratively normalizing rows ($\mathcal{R}$) and columns ($\mathcal{C}$) $\ell$ times. It can be shown [1] that $S(X) := \lim_{\ell\to\infty} S^\ell(X)$ defines a smooth operator mapping to bistochastic matrices[1] and that, for almost all $X$,

$$\lim_{\tau\to 0^+} S(X/\tau) = M(X) := \arg\max_{P\in\mathcal{P}_N}[\mathrm{trace}(P^{\mathrm{T}}X)]. \tag{S2}$$

The operator $M$ defined in Eq. (S2), which maps onto permutation matrices, can be computed using standard discrete algorithms for linear assignment problems [2]. Eq. (S2) implies that, using the "parameterization matrices" $X$, one can smoothly navigate the space $\mathcal{B}_N$ of all $N \times N$ bistochastic (resp. near-bistochastic) matrices using $S$ (resp. $S^\ell$), while keeping track of the "nearest" permutation matrices using $M$. In what follows, we will refer to (near-)bistochastic matrices as "soft permutations" and to true permutations as "hard permutations".

We may therefore hope to find optimal hard permutations for the original score $\mathscr{S}$ by optimizing a suitable smooth extension $\hat{\mathscr{S}}$ of $\mathscr{S}$ to soft permutations, since this can be done efficiently using gradient methods. In general, optimization of $\hat{\mathscr{S}}$ can be very sensitive to hyperparameters such as the "temperature" $\tau > 0$ in Eq. (S2), the standard deviation of the entries of $X$ at initialization, the optimizer learning rate, and the strength of regularization.

## 1.2 Initialization and bootstrapped optimization

We found that solving $X^* = \arg\min_X \hat{\mathscr{L}}_{2\mathrm{BE}}(S^\ell(X/\tau))$, for some choice of $\ell$ and $\tau$, using several steps of gradient descent, generally yields sub-optimal hard permutations $M(X^*)$ for the original loss $\mathscr{L}_{2\mathrm{BE}}$, see Eq. (S2). Informally, this is because hard permutations are local minima for $\hat{\mathscr{L}}$, a situation reminiscent of how the entropy of a single Bernoulli random variable with parameter $p$ has minima at the "sharp" cases $p = 0$ and $p = 1$. Nevertheless, we found that outcomes improved when all entries of $X$ are initialized to be zero. Indeed, as shown in Fig. S1 for the benchmark HK-RR prokaryotic dataset described in Supplementary material Section 2, the *first* gradient step alone, when $X \equiv 0$ at initialization, is often competitive with a full-blown discrete algorithm for approximate maximization of $\mathscr{S}_{\mathrm{MI}}$, called MI-IPA [3]. Finally, as previously observed using several methods [3–6], pairing performance can be expected to increase if correct pairings are used as fixed context, biasing the computation of the two-body entropies. Together, these considerations led us to DiffPaSS, our proposed bootstrapped approach to differentiable pairing. Given a loss $\mathscr{L}$ and its smooth extension $\hat{\mathscr{L}}$, two ordered collections $\mathcal{M}_{\mathrm{A}}$ and $\mathcal{M}_{\mathrm{B}}$ containing $D$ sequences, and (optionally) a pre-existing set $\mathcal{F}_{\mathrm{AB}} = \{(a_i, b_i)\}_{i=1}^{D_{\mathrm{fix}}}$ containing $D_{\mathrm{fix}}$ matched pairs of sequences, we proceed as follows.[2] Let $\Delta n$ be a positive integer, referred to in the main text as the step size, and $N_{\mathrm{steps}} \geq 1$ be such that $(N_{\mathrm{steps}}-1)\Delta n$ is the largest multiple of $\Delta n$ that is less than $D - D_{\mathrm{fix}}$. Initialize $\mathcal{F}' = \mathcal{F}_{\mathrm{AB}}$ and $n_{\mathrm{prev}} = 0$; then, for every $n = \Delta n, 2\Delta n, \ldots, (N_{\mathrm{steps}} - 1)\Delta n, D - D_{\mathrm{fix}}$:

1. if $n_{\mathrm{prev}} = D - D_{\mathrm{fix}}$, terminate;
2. define $P'$ as the $(D_{\mathrm{fix}}+n_{\mathrm{prev}})\times(D_{\mathrm{fix}}+n_{\mathrm{prev}})$ permutation matrix corresponding to the matchings in $\mathcal{F}'$;
3. initialize two zero $D \times D$ matrices $P$ and $\hat{P}$, and copy $P'$ into the row-column pairs belonging to $\mathcal{F}'$ in both cases;
4. initialize a $(D - D_{\mathrm{fix}} - n_{\mathrm{prev}}) \times (D - D_{\mathrm{fix}} - n_{\mathrm{prev}})$ parameterization matrix $X \equiv 0$, to be used for sequences not involved in pairs in $\mathcal{F}'$;
5. update $X \leftarrow -\nabla(\hat{\mathscr{L}} \circ \tilde{S}^\ell)(X = 0)$, where $\tilde{S}^\ell(X)$ denotes copying $S^\ell(X)$ into the submatrix of $\hat{P}$ obtained by removing rows and columns involved in pairs in $\mathcal{F}'$;

[1] A bistochastic matrix is a matrix with non-negative entries whose all rows and columns sum to 1.
[2] An animation illustrating this algorithm in the special case $D_{\mathrm{fix}} = 0$ is available at the following URL: https://www.youtube.com/watch?v=G2rV4ldgTIY.

6. compute the hard permutation $\pi = \tilde{M}(X)$, where $\tilde{M}(X)$ denotes copying $M(X)$ into the submatrix of $P$ obtained by removing rows and columns involved in pairs in $\mathcal{F}'$;
7. pick $n$ pairs of sequences matched by $\pi$, but not in $\mathcal{F}_{\mathrm{AB}}$, uniformly at random;
8. update $\mathcal{F}' \leftarrow \mathcal{F}_{\mathrm{AB}} \cup \{\text{pairs selected in step 7}\}$;
9. update $n_{\mathrm{prev}} \leftarrow n$.

For every $n = \Delta n, 2\Delta n, \ldots, (N_{\mathrm{steps}} - 1)\Delta n, D - D_{\mathrm{fix}}$, we record the loss $\mathscr{L}(\pi)$ at step 6, and the final output of DiffPaSS is the hard permutation $\pi^*$ corresponding to the lowest recorded loss.

Note that the gradient of $S^\ell(X/\tau)$, evaluated at $X = 0$, only changes by a global scale factor if $\tau$ is changed, and the matching operator $M$ is scale-invariant. Hence, we can set $\tau = 1$ as the obtained hard permutations are independent of it. Similarly, all hard permutations obtained are independent of the choice of learning rate and regularization strength. Perhaps more surprisingly, for any $\ell > 1$, the gradient of $S^\ell$ evaluated at $X = 0$ is equal to corresponding gradient of $S^{\ell=1}$. We prove this in Supplementary material Section 1.3. Hence, we can set $\ell = 1$ throughout, leading to significant runtime gains.

**Robust pairs and DiffPaSS-IPA.** We noticed that, even when the starting set $\mathcal{F}_{\mathrm{AB}}$ of fixed pairs is empty, some pairs are matched by all the $N_{\mathrm{steps}}$ hard permutations $\pi$ explored by DiffPaSS (see step 6 in Supplementary material Section 1.2). We call these *robust pairs*, and notice that they tend to have high precision, see Fig. S2. This suggests that they can be used as the starting set $\mathcal{F}_{\mathrm{AB}}$ of fixed pairs in a further run of DiffPaSS. We call this procedure DiffPaSS-IPA ("Iterative Pairing Algorithm", following Bitbol [3], Bitbol et al. [4]). It can be iterated several times, enlarging the set of robust pairs after each iteration, or stopping if no further robust pairs are found. The final output of DiffPaSS-IPA is the hard permutation with lowest observed loss across all IPA runs. We use $N_{\mathrm{IPA}} = 3$ iterations throughout.

### 1.3 Independence of number of Sinkhorn normalizations

Let $S^\ell$ (for an integer $\ell > 0$) be the $\ell$-truncated Sinkhorn operator defined in Eq. (S1). In this section we prove that, for any $\ell > 1$, all first-order derivatives of $S^\ell$, when evaluated at the zero matrix, are equal to the corresponding derivatives of $S^{\ell=1}$.

Let $\mathcal{R}$ (resp. $\mathcal{C}$) be the row-wise (resp. column-wise) matrix normalization operator on $D \times D$ matrices. Denote the partial derivative operator with respect to the $(i, j)$-th matrix entry by $\partial_{ij}$, and the $(k, l)$-th matrix component of a matrix-valued operator $\mathcal{O}$ by $\mathcal{O}_{kl}$. Furthermore, let $\mathcal{T}^* = \mathcal{C} \circ \mathcal{R}$. Let $\mathbf{1}_{\mathrm{mat}}$ denote the $D \times D$ matrix whose entries are all equal to 1. Given the definition of the Sinkhorn operator in Eq. (S1), and since the componentwise exponential of the zero matrix is a matrix of ones, it suffices, for our purposes, to show that

$$[\partial_{ij}(\mathcal{T}^* \circ \mathcal{T}^*)_{kl}](\mathbf{1}_{\mathrm{mat}}) = [\partial_{ij}\mathcal{T}^*_{kl}](\mathbf{1}_{\mathrm{mat}}) \tag{S3}$$

for all $i, j, k, l = 1, \ldots, D$. Indeed, we will prove that this actually holds when both sides of Eq. (S3) are evaluated at $\mu\mathbf{1}_{\mathrm{mat}}$, for any real number $\mu > 0$.

*Proof of Eq. (S3).* Let $\boldsymbol{X}$ denote a $D \times D$ matrix with positive entries. We begin by noting that

$$[\partial_{ij}\mathcal{R}_{kl}](\boldsymbol{X}) = \delta_{ik}\,[\partial_j \mathcal{T}_l](\boldsymbol{X}_{i\cdot}) \quad \text{and} \quad [\partial_{ij}\mathcal{C}_{kl}](\boldsymbol{X}) = \delta_{jl}\,[\partial_i \mathcal{T}_k](\boldsymbol{X}_{\cdot j})\,, \tag{S4}$$

where $\delta$ denotes the Kronecker delta, $\mathcal{T}$ the normalization operator for $D$-dimensional vectors, and $\boldsymbol{X}_{i\cdot}$ (resp. $\boldsymbol{X}_{\cdot j}$) the $i$-th row (resp. $j$-th column) of $\boldsymbol{X}$.

Let $\boldsymbol{x}$ denote a $D$-dimensional vector. The partial derivatives of the components of $\mathcal{T}$, evaluated at $\boldsymbol{x}$, are given by

$$\partial_\alpha \mathcal{T}_\beta(\boldsymbol{x}) = \frac{\partial}{\partial \alpha} \frac{x_\beta}{\sum_\gamma x_\gamma} = \frac{\delta_{\alpha\beta}}{\sum_\gamma x_\gamma} - \frac{x_\beta}{(\sum_\gamma x_\gamma)^2}. \tag{S5}$$

Let $\mathbf{1}$ denote the $D$-dimensional vector whose entries are all equal to 1. Applying Eq. (S5) to $\boldsymbol{x} = \mu\mathbf{1}$ yields, for any real number $\mu > 0$,

$$\partial_\alpha \mathcal{T}_\beta(\mu\mathbf{1}) = \frac{1}{D\mu}(\delta_{\alpha\beta} - 1/D) = \frac{1}{D\mu}\mathbf{\Delta}_{\alpha\beta}, \tag{S6}$$

where we have defined the square matrix $\mathbf{\Delta} = \mathrm{Id} - \mathbf{1}_{\mathrm{mat}}/D$, with Id denoting the $D \times D$ identity matrix. We remark for later that $\mathbf{1}_{\mathrm{mat}}/D$ is idempotent (i.e., its matrix square is itself), and that therefore so is $\mathbf{\Delta}$.

Using the chain rule and the fact that $\mathcal{R}(\mu\mathbf{1}_{\mathrm{mat}}) = \mathbf{1}_{\mathrm{mat}}/D$ for any $\mu > 0$, we can write

$$\begin{aligned}
[\partial_{ij}\mathcal{T}^*_{kl}](\mu\mathbf{1}_{\mathrm{mat}}) &= \sum_{m,n}[\partial_{mn}\mathcal{C}_{kl}](\mathbf{1}_{\mathrm{mat}}/D)\,[\partial_{ij}\mathcal{R}_{mn}](\mu\mathbf{1}_{\mathrm{mat}}) \\
&= \sum_{m,n}\delta_{nl}\mathbf{\Delta}_{mk}\,\frac{\delta_{im}}{D\mu}\mathbf{\Delta}_{jn} \\
&= \frac{1}{D\mu}\mathbf{\Delta}_{ik}\mathbf{\Delta}_{jl}\,,
\end{aligned} \tag{S7}$$

where we used Eq. (S4) and Eq. (S6) to obtain the second line.

We now compute

$$\begin{aligned}
[\partial_{ij}(\mathcal{T}^* \circ \mathcal{T}^*)_{kl}](\mu\mathbf{1}_{\mathrm{mat}}) &= \sum_{mn}[\partial_{mn}\mathcal{T}^*_{kl}](\mathbf{1}_{\mathrm{mat}}/D)\,[\partial_{ij}\mathcal{T}^*_{mn}](\mu\mathbf{1}_{\mathrm{mat}}) \\
&= \frac{1}{D\mu}\sum_{mn}\mathbf{\Delta}_{mk}\mathbf{\Delta}_{nl}\mathbf{\Delta}_{im}\mathbf{\Delta}_{jn} \\
&= \frac{1}{D\mu}(\mathbf{\Delta}^2)_{ik}(\mathbf{\Delta}^2)_{jl} \\
&= [\partial_{ij}\mathcal{T}^*_{kl}](\mu\mathbf{1}_{\mathrm{mat}}),
\end{aligned}$$

where the last equality follows from the idempotency of $\mathbf{\Delta}$ and from Eq. (S7). When $\mu = 1$, this proves Eq. (S3). □

# 2 Datasets

**Benchmark prokaryotic datasets.** We developed and tested DiffPaSS using joint MSAs extracted from a dataset composed of 23,632 cognate pairs of histidine kinases (HK) and response regulators (RR) from the P2CS database [7, 8], paired using genome proximity, and previously described in Bitbol [3], Bitbol et al. [4]. Our focus is on pairing interaction partners among paralogs within each species. Pairing is trivial for a small number of species comprising only one pair of sequences. Hence, these species were discarded from the dataset. The average number of pairs per species in the resulting dataset is 11.0.

From this benchmark dataset of known interacting pairs, we extract paired MSAs of average depth 50, 100, 200, 300, 400, 500, 750 or 1000, constructed by selecting all the sequences of randomly sampled species from the full dataset. Each depth bin contains at least 200 MSAs. More precisely, for a target MSA depth $\overline{D} = 50$, 100, 200, 300, 400, 500, 750 or 1000, we add randomly sampled complete species one by one; if the first $m$ species (but no fewer) give an MSA depth $D \geq 0.9\overline{D}$, and the first $n \geq m$ species (but no more) give $D \leq 1.1\overline{D}$, then we select the first $k$ species in our final MSA, with $k$ picked uniformly at random between $m$ and $n$.

**Eukaryotic complexes.** We consider 15 heteromeric eukaryotic targets whose structures are not in the training set of AFM with v2 weights, already considered in Lupo et al. [6].

**T-cell receptor paired CDR3$\alpha$-CDR3$\beta$ data.** We downloaded the full VDJDb database [9] and removed all entries where only a single TCR chain is available. For each epitope, we removed duplicate TCRs (defined at the level of $\alpha/\beta$) and retained only epitopes for which at least 100 and no more than 10,000 sequences were available. Patient and study metadata from the database is used to define "groups" that permutations are to be restricted to, and that play the part of species in our paralog matching problem. Our final dataset contains 22 sets of CDR3$\alpha$-CDR3$\beta$ sequence collections of highly variable total size (ranging from 103 to 1894 sequence pairs) and mean group size, for which the ground-truth matchings are known.

# 3 General points on AlphaFold-Multimer (AFM)

For all structure prediction tasks, we use the five pre-trained AFM models with v2 weights [10]. We use full genomic databases and code from release v2.3.1 of the official implementation in `https://github.com/deepmind/alphafold`. We use no structural templates, and perform 3 recycles for each structure, without early stopping. We relax all models using AMBER.

We pair the same subset of pairable sequence retrieved by AFM as Lupo et al. [6], and refer to Lupo et al. [6, Table S1] for details on the pairable MSAs. For all structures, we use the query protein pair as fixed context for DiffPaSS.

The AFM confidence score is defined as $0.8 \cdot \text{iptm} + 0.2 \cdot \text{ptm}$, where iptm is the predicted TM-score in the interface, and ptm the predicted TM-score of the entire complex [10].

# 4 Supplementary figures

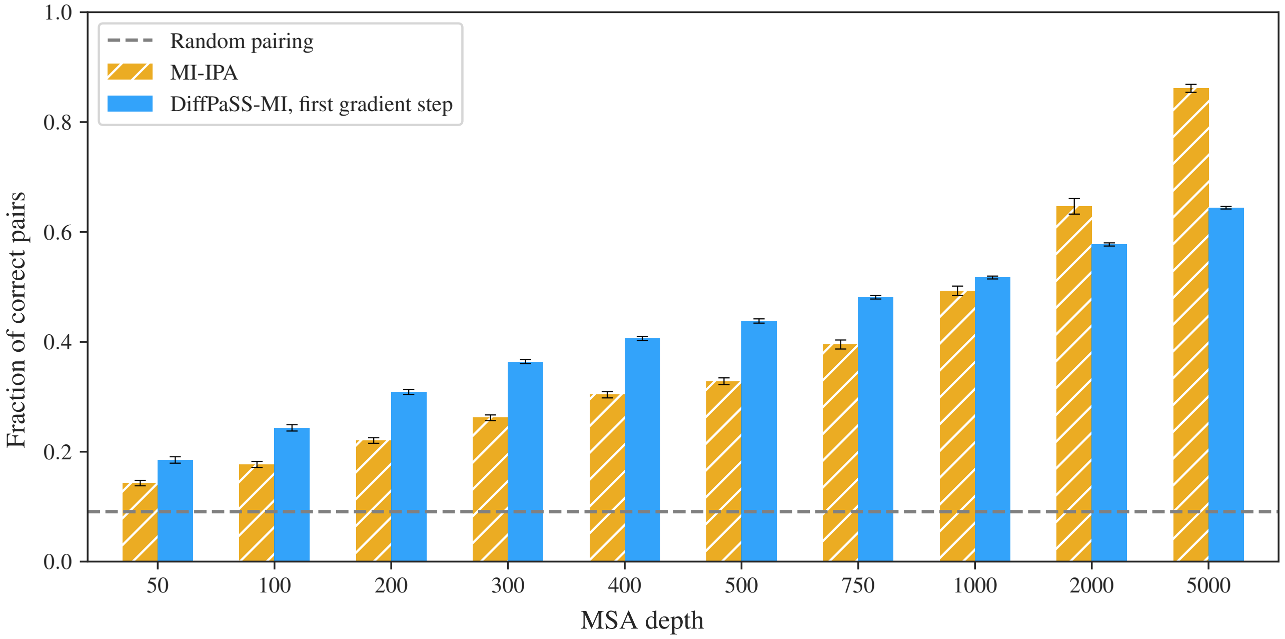


Figure S1: **DiffPaSS pairing performance after the first gradient step.** Pairing performance by DiffPaSS is shown after the first gradient step and compared to MI-IPA [3] on HK-RR MSAs with various depths. Same as in Fig. 2 but restricting to the first gradient step. Here, our procedure, after only one gradient step, is competitive with MI-IPA [3], for approximate maximization of the same discrete score $\mathscr{S}_{\text{MI}}$.

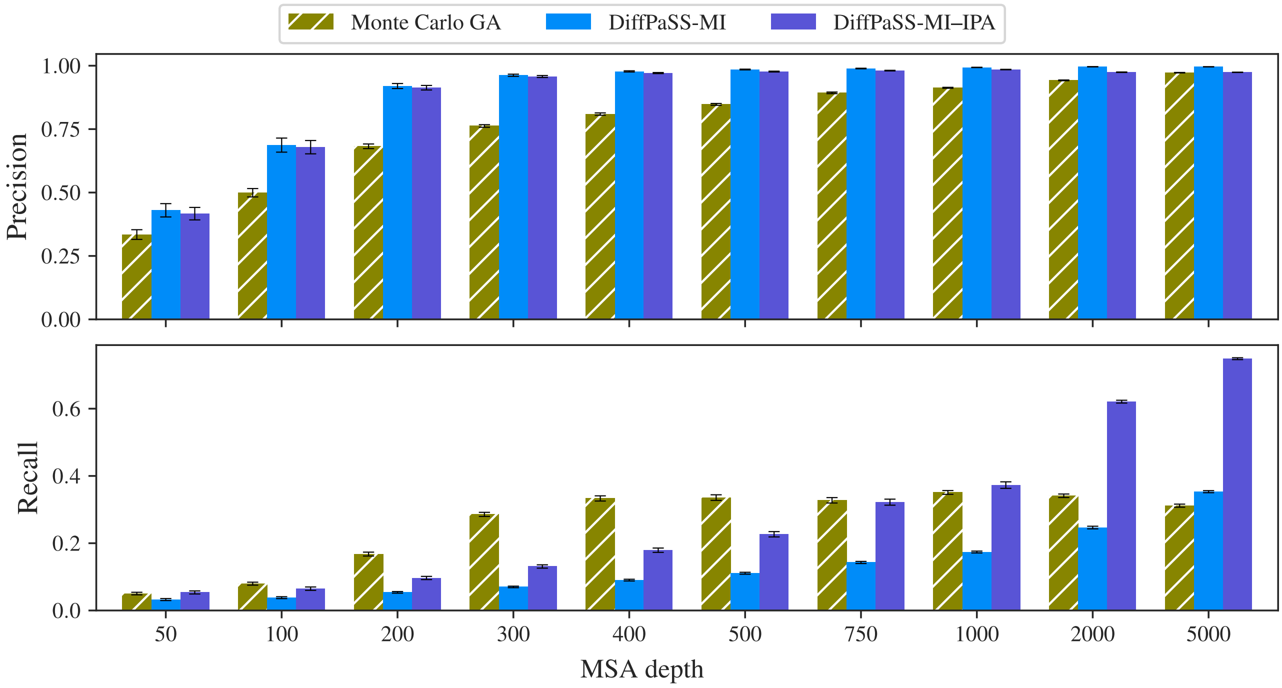


Figure S2: **Robust pairs.** Precision and recall for the robust pairs found by the Monte Carlo GA plus MI-IPA method (using 20 independent GA runs, as in [5]), DiffPaSS-MI, and DiffPaSS-MI–IPA with $N_{\mathrm{IPA}} = 3$ for several MSA depths, on the HK-RR dataset. Same MSAs as in Fig. 2.

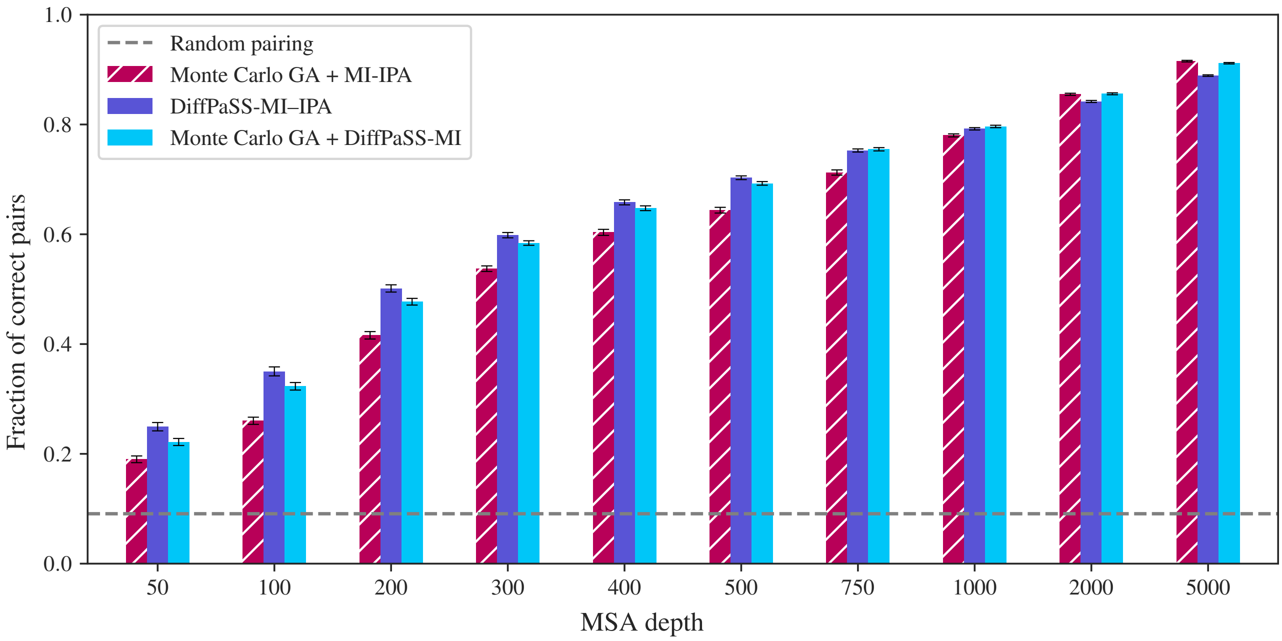


Figure S3: **Combining DiffPaSS-MI and Monte Carlo GA.** The performance of DiffPaSS-MI combined with Monte Carlo GA is evaluated on the same MSAs as in Fig. 2, starting from the same robust pairs identified by Monte Carlo GA. For comparison, we reproduce the bars showing the performance of DiffPaSS-MI–IPA alone, and of MI-IPA combined with Monte Carlo GA, from Fig. 2.

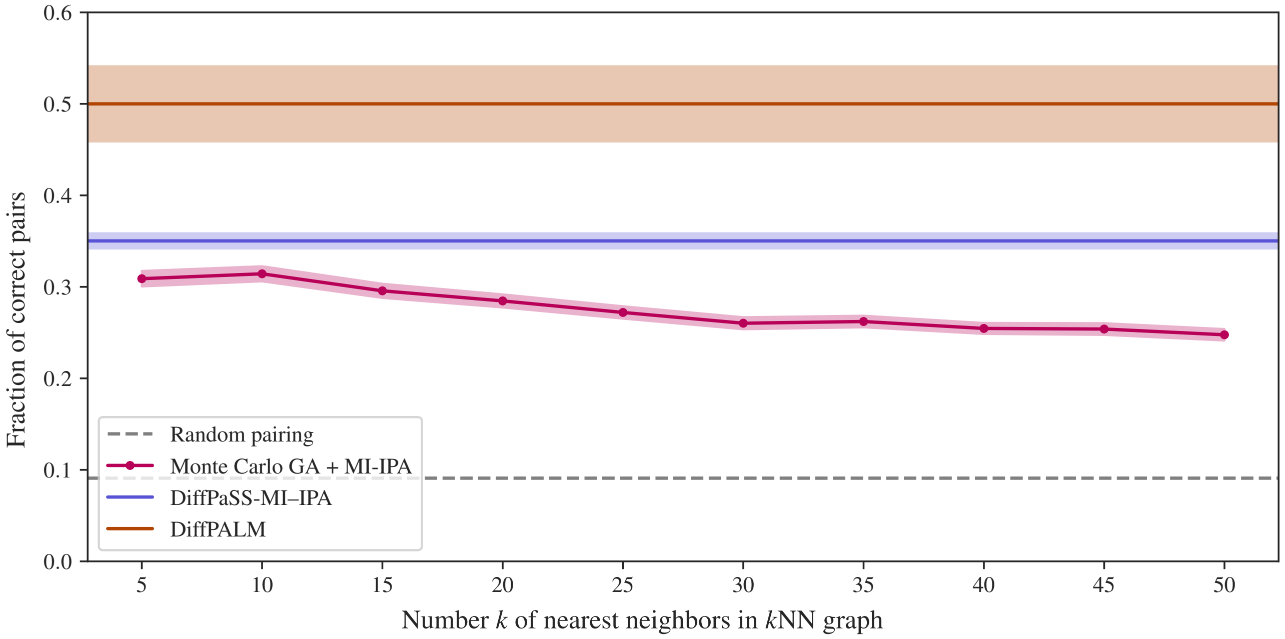


Figure S4: **Detailed comparison of MI-IPA combined with Monte Carlo GA with DiffPaSS-MI–IPA and DiffPALM, for shallow HK-RR MSAs.** For the same HK-RR MSAs of depth 100 as in Fig. 2, we study the impact of the number $k$ of nearest neighbors in the $k$NN graph considered for Monte Carlo GA, see [5], on the performance of MI-IPA combined with Monte Carlo GA [5]. The performances of DiffPaSS-MI–IPA and of DiffPALM for the same datasets are shown for comparison. Recall that they do not depend on $k$ since the $k$NN graph is not used in these methods.

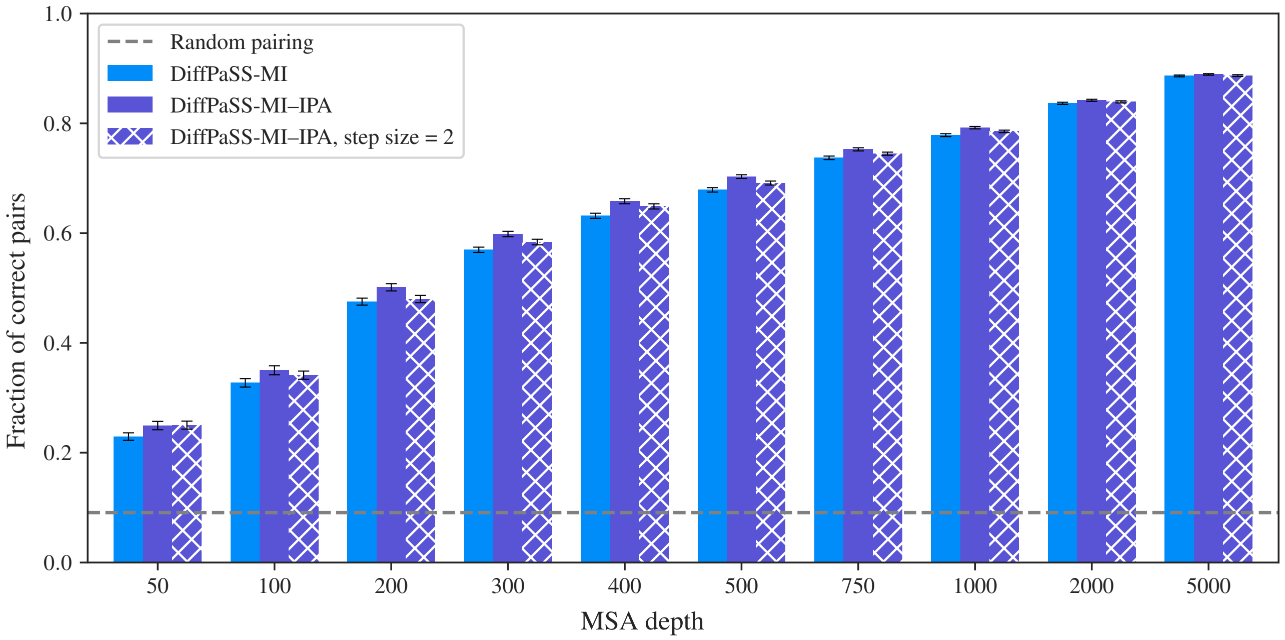


Figure S5: **Impact of the step size $\Delta n$ on DiffPaSS-MI–IPA.** The performance of DiffPaSS-MI–IPA with step size 2 (i.e. when increasing by 2 the number of fixed pairs at each optimization run of DiffPaSS) is compared to that of DiffPaSS-MI–IPA with step size 1 (our default choice), and to that of DiffPaSS-MI, for HK-RR MSAs with different depths. Results for the latter two methods, shown for comparison, are identical to those in Fig. 2. The MSAs and the performance metrics used are the same as in Fig. 2.

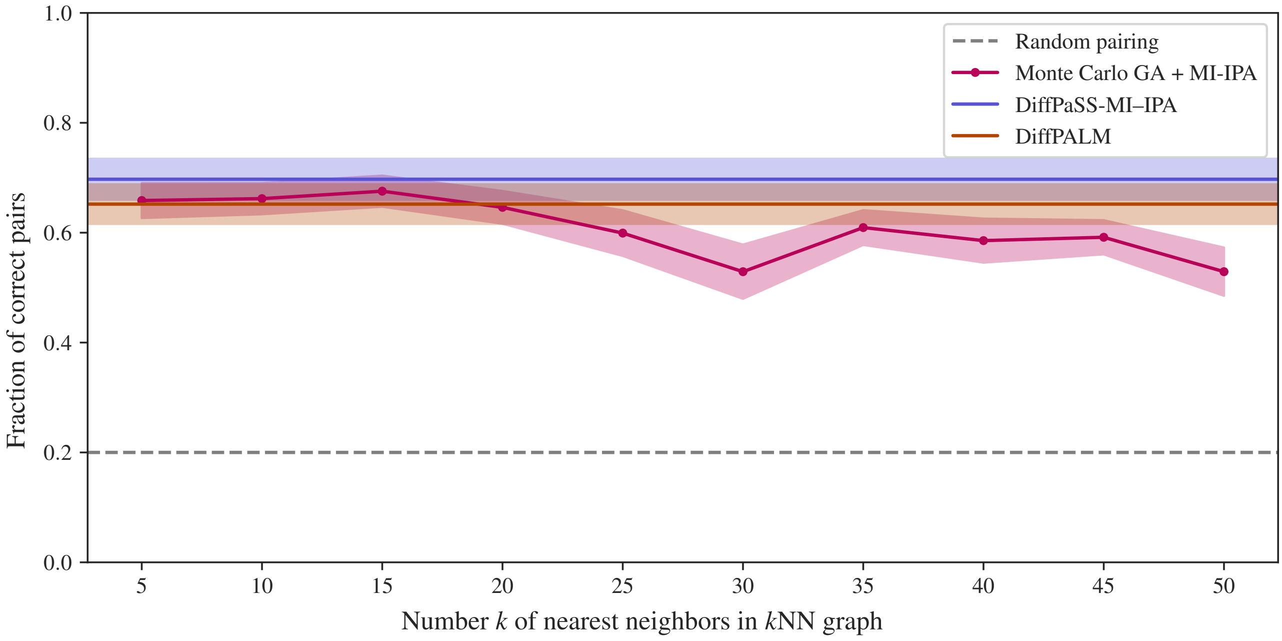


Figure S6: **Comparison of DiffPaSS-MI–IPA with DiffPALM and with MI-IPA combined with Monte Carlo GA for MALG-MALK.** Same as Fig. S4, but for the same MALG-MALK MSAs of depth 100 as in Lupo et al. [6].

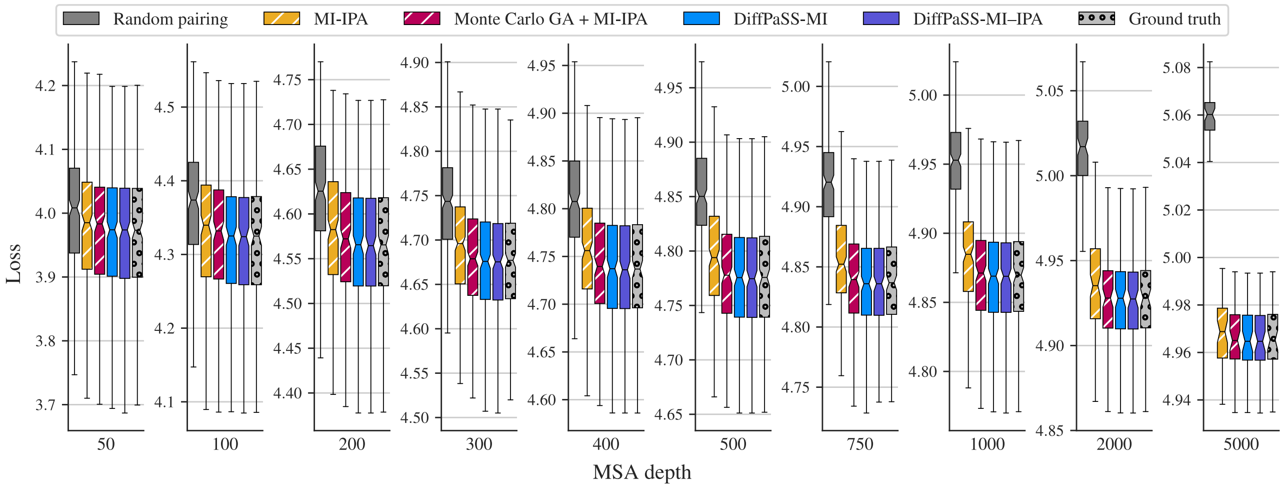


Figure S7: **Inter-chain two-body entropy losses for random, predicted and ground-truth pairings.** The distributions of the inter-chain two-body entropy losses are shown for several methods and several MSA depths, on the HK-RR dataset. Same MSAs as in Fig. 2.

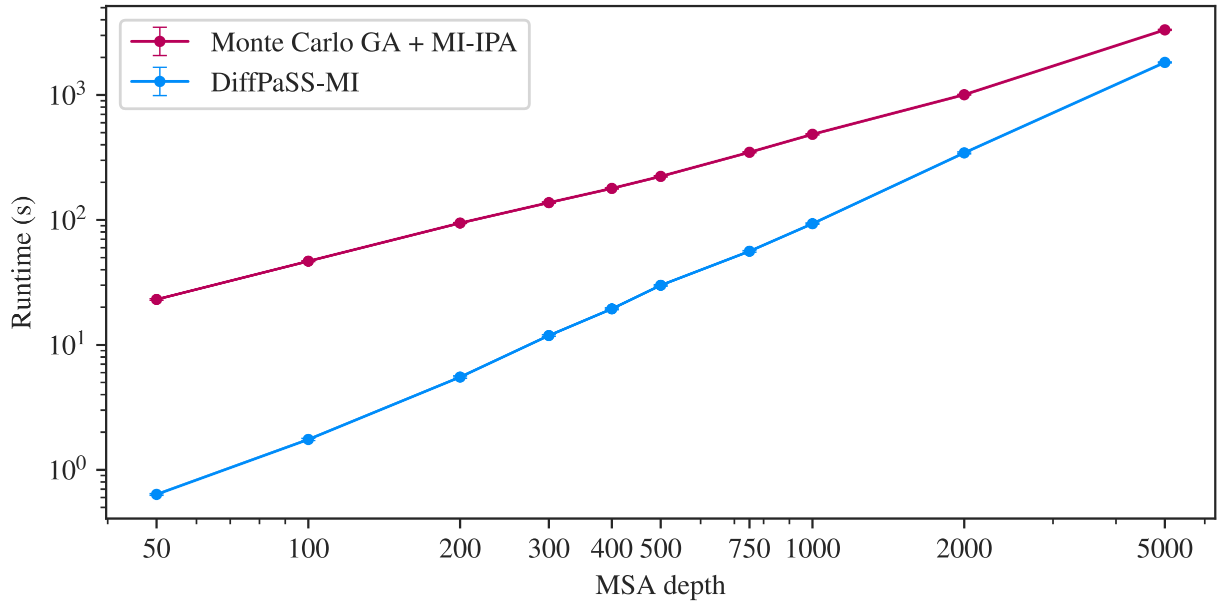


Figure S8: **Computational runtime.** Runtime comparison between DiffPaSS-MI and Monte Carlo GA + MI-IPA [5], on the same HK-RR MSAs as in Fig. 2. DiffPaSS was implemented in `PyTorch` v2.2.1, and run on an NVIDIA® GeForce RTX™ A6000 GPU. We used the original implementation of the combined Monte Carlo GA and MI-IPA method, in the Julia programming language (Julia v1.10.0). All 20 GA replicates were run in parallel on separate Intel® Xeon® Platinum 8360Y CPUs running at 2.4 GHz and then MI-IPA was run on one of these CPUs.

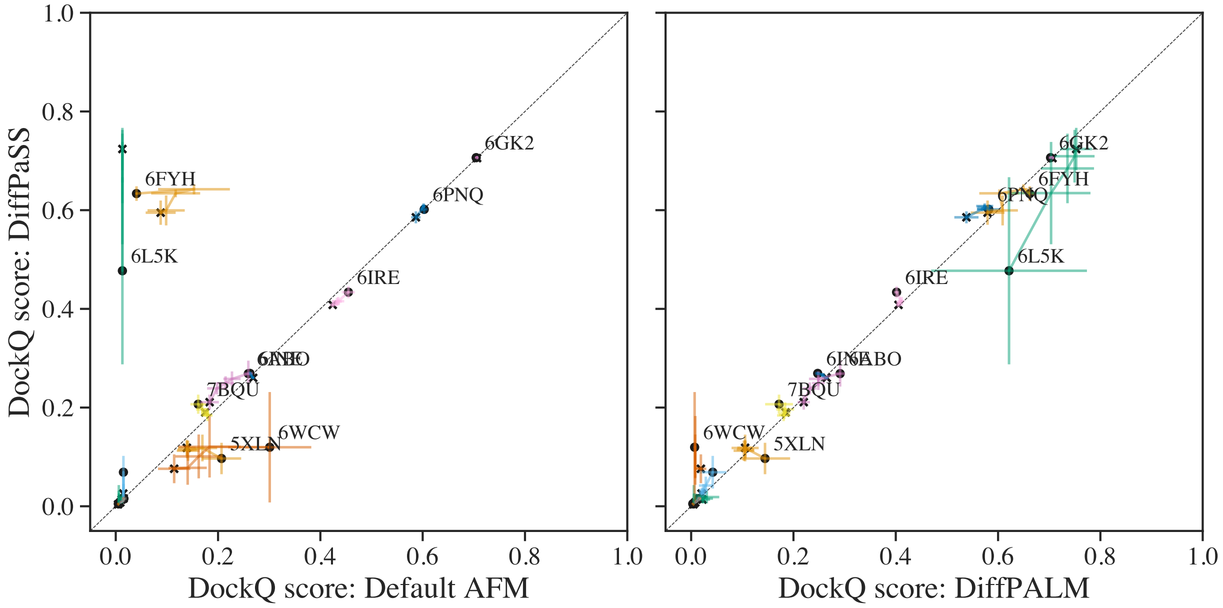


Figure S9: **Performance of structure prediction by AFM using different MSA pairing methods.** We report the performance of AFM, in terms of DockQ scores, for the 15 complexes considered in [6], using different pairing methods on the same MSAs. Left panel: DiffPaSS versus default AFM pairing. Right panel: DiffPaSS versus DiffPALM. For each complex, AFM is run five times, and the five top predicted structures by AFM confidence are considered each time, yielding 25 predicted structures in total. For each complex, we show "trajectories" of performance starting from the top-confidence predicted structure (black circular marker) and ending with all predicted structures up to and including the fifth one (black cross marker). Results are averaged over the 5 runs and standard errors are shown as error bars. Points with DockQ below 0.1 are not labelled with their PDB ID for graphical reasons.

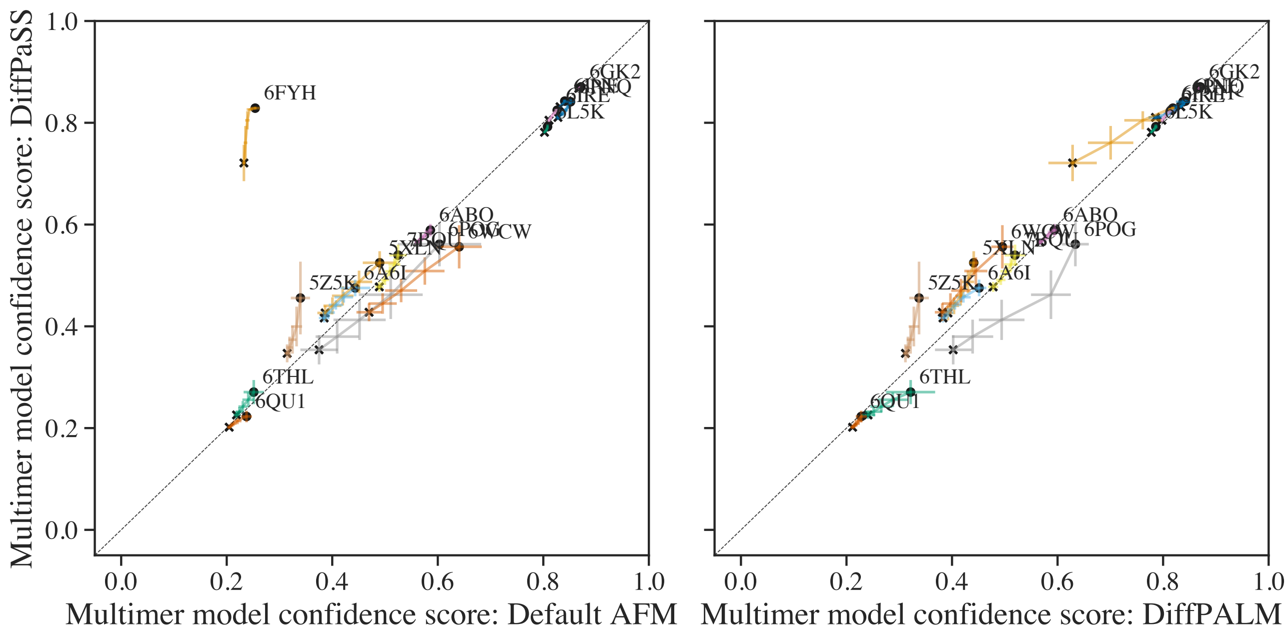


Figure S10: **AlphaFold-Multimer confidence scores on eukaryotic complexes using different MSA pairing methods.** Same comparisons as in Fig. S9, but showing the confidence scores for structure prediction by AFM instead of DockQ scores. See Supplementary material Section 3 for a definition of this confidence score. Results for Default AFM and DiffPALM-based pairing are as in Lupo et al. [6, Fig. S5].

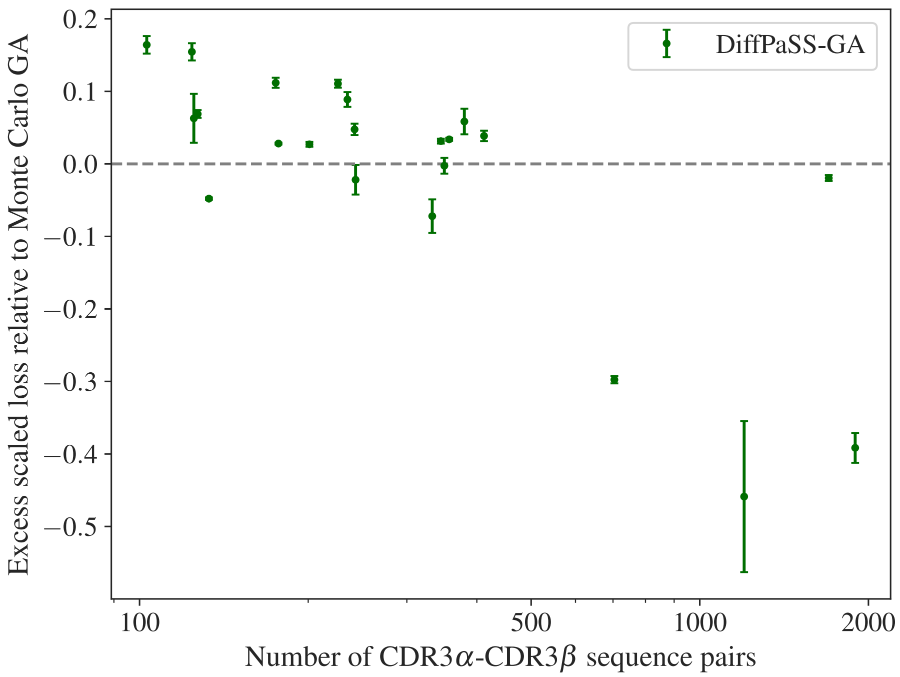


Figure S11: **DiffPaSS optimization and pairing quality on the CDR3$\alpha$-CDR3$\beta$ dataset.** (Normalized) graph alignment scores per epitope were computed in the paired CDR3$\alpha$-CDR3$\beta$ dataset described in Supplementary material Section 2 with both DiffPaSS-GA and the Monte Carlo GA from Gandarilla-Pérez et al. [5]. We plot the difference between the loss obtained by DiffPaSS-GA and by Monte Carlo GA versus the number of pairs for each epitope. Negative values indicate that Monte DiffPaSS-GA achieves a more successful optimization. Each point represents one of the epitopes in the dataset. Markers show mean values across 20 runs, and error bars are defined as $\sqrt{\sigma^2_{\text{MC}} + \sigma^2_{\text{DiffPaSS}}}$, where $\sigma_{\text{MC}}$ (resp. $\sigma_{\text{DiffPaSS}}$) is the standard deviation obtained by Monte Carlo GA (resp. DiffPass-GA). Graph alignment scores are as defined in [5], but normalized by the number of CDR3$\alpha$ and CDR3$\beta$ sequences to pair in each case.

## References


[1] Gonzalo E. Mena, David Belanger, Scott Linderman, and Jasper Snoek. Learning latent permutations with Gumbel-Sinkhorn networks. *6th International Conference on Learning Representations, ICLR 2018 - Conference Track Proceedings*, pages 1–22, 2018. URL https://openreview.net/forum?id=Byt3oJ-0W.

[2] H. W. Kuhn. The Hungarian method for the assignment problem. *Naval Research Logistics Quarterly*, 2:83–97, 1955. doi: 10.1002/nav.3800020109.

[3] Anne-Florence Bitbol. Inferring interaction partners from protein sequences using mutual information. *PLoS Comput. Biol.*, 14(11):e1006401, 2018. doi: 10.1371/journal.pcbi.1006401.

[4] Anne-Florence Bitbol, Robert S Dwyer, Lucy J Colwell, and Ned S Wingreen. Inferring interaction partners from protein sequences. *Proc. Natl. Acad. Sci. U.S.A.*, 113(43):12180–12185, 2016. doi: 10.1073/pnas.1606762113.

[5] Carlos A. Gandarilla-Pérez, Sergio Pinilla, Anne-Florence Bitbol, and Martin Weigt. Combining phylogeny and coevolution improves the inference of interaction partners among paralogous proteins. *PLoS Comput. Biol.*, 19(3):e1011010, 2023. doi: 10.1371/journal.pcbi.1011010.

[6] Umberto Lupo, Damiano Sgarbossa, and Anne-Florence Bitbol. Pairing interacting protein sequences using masked language modeling. *Proceedings of the National Academy of Sciences*, 121 (27):e2311887121, 2024. doi: 10.1073/pnas.2311887121. URL https://www.pnas.org/doi/abs/10.1073/pnas.2311887121.

[7] M. Barakat, P. Ortet, C. Jourlin-Castelli, M. Ansaldi, V. Mejean, and D. E. Whitworth. P2CS: a two-component system resource for prokaryotic signal transduction research. *BMC Genomics*, 10:315, 2009. doi: 10.1186/1471-2164-10-315.

[8] M. Barakat, P. Ortet, and D. E. Whitworth. P2CS: a database of prokaryotic two-component systems. *Nucleic Acids Research*, 39(Database issue):D771–776, 2011. doi: 10.1093/nar/gkq1023.

[9] Mikhail Goncharov, Dmitry Bagaev, Dmitrii Shcherbinin, Ivan Zvyagin, Dmitry Bolotin, Paul G. Thomas, Anastasia A. Minervina, Mikhail V. Pogorelyy, Kristin Ladell, James E. McLaren, David A. Price, Thi H.O. Nguyen, Louise C. Rowntree, E. Bridie Clemens, Katherine Kedzierska, Garry Dolton, Cristina Rafael Rius, Andrew Sewell, Jerome Samir, Fabio Luciani, Ksenia V. Zornikova, Alexandra A. Khmelevskaya, Saveliy A. Sheetikov, Grigory A. Efimov, Dmitry Chudakov, and Mikhail Shugay. VDJdb in the pandemic era: a compendium of T cell receptors specific for SARS-CoV-2. *Nature Methods 2022 19:9*, 19(9):1017–1019, August 2022. ISSN 1548-7105. doi: 10.1038/s41592-022-01578-0. URL https://www.nature.com/articles/s41592-022-01578-0. Publisher: Nature Publishing Group.

[10] Richard Evans, Michael O'Neill, Alexander Pritzel, Natasha Antropova, Andrew Senior, Tim Green, Augustin Žídek, Russ Bates, Sam Blackwell, Jason Yim, Olaf Ronneberger, Sebastian Bodenstein, Michal Zielinski, Alex Bridgland, Anna Potapenko, Andrew Cowie, Kathryn Tunyasuvunakool, Rishub Jain, Ellen Clancy, Pushmeet Kohli, John Jumper, and Demis Hassabis. Protein complex prediction with AlphaFold-Multimer. *bioRxiv*, 2021. doi: 10.1101/2021.10.04.463034.
